# Contextual Semantic Pattern Recognition for Mental Health Monitoring Using ClinicalBERT Transformers

**DOI:** 10.1002/brb3.71879

**Published:** 2026-11-02

**Authors:** Junjun Sun

**Affiliations:** ^1^ School of Foreign Languages Shandong Vocational and Technical University of International Studies Rizhao Shandong China

**Keywords:** ClinicalBERT, contextual semantic representation, deep learning, explainable artificial intelligence, mental health monitoring, Transformers

## Abstract

**Purpose:**

This study aimed to develop an interpretable ClinicalBERT‐based framework for mental health monitoring using structured clinical data.

**Method:**

Because the Mental Health Diagnosis and Treatment Monitoring dataset contains structured patient attributes rather than free‐text clinical narratives, a structured‐to‐text transformation module was used to convert the records into contextual descriptions suitable for ClinicalBERT. The proposed model was compared with Random Forest, XGBoost, Bidirectional Encoder Representations from Transformers (BERT), TabTransformer, and a Deep Neural Network, and to strengthen the experimental comparison, contemporary tabular transformer models, including SAINT and FT‐Transformer, were also evaluated. Model performance was assessed using accuracy, precision, recall, and F1‐score. Cross‐validation, confidence interval analysis, statistical significance testing, and ablation experiments were conducted to examine the reliability of the results. SHAP and LIME were used to explain global feature contributions and individual predictions.

**Finding:**

ClinicalBERT achieved an accuracy of 90.2%, a precision of 90.5%, a recall of 90.4%, and an F1‐score of 90.6%, outperforming all comparison models. The additional validation analyses supported the stability of the results. SHAP and LIME further identified the patient attributes that contributed most strongly to the model's predictions.

**Conclusion:**

The findings indicate that structured clinical records can be transformed into contextual representations for transformer‐based mental health monitoring. The framework combines predictive performance with interpretable output, although its evaluation is currently limited to a single dataset. Further validation using multi‐institutional and prospective data is required.

## Graphical abstract

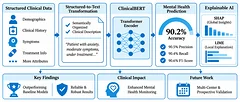

## 1 Introduction

Mental health is now a worldwide issue, and depression, anxiety, stress, and associated mental health problems remain a problem for people in various age groups and communities around the world (Haldorai et al. 2024). The typical approach to mental health assessment is through clinical interviews, self‐reported data, and periodic monitoring, all of which can be limited in their ability to assess, be delayed, or suffer from the varying nature of patient conditions (Khan et al. 2025a). As a result, the use of artificial intelligence (AI) and deep learning (DL) has been studied extensively to automatically extract mental‐health‐related information and to extract complex patterns from demographic, behavioral, and clinical data (Karimi et al. 2023).

Recent developments in the transformer‐based learning paradigm have opened new avenues for learning contextual representations from health‐related information (Priya Sri et al. 2021). However, many mental‐health monitoring datasets are only available in structured form, which includes attributes like age, sex, diagnosis, severity of symptoms, mood score, quality of sleep, level of physical activity, medication, form of therapy, and outcomes of treatment (Meng et al. 2024). Such parameters are easily handled by conventional machine‐learning and tabular deep‐learning models but can be problematic for capturing contextual relationships between the different types of attributes (Pourkeyvan et al. 2024). Thus, a key methodological issue is to construct a representation strategy that retains the original structure of the information maintained in such a way that contextual semantic learning can be achieved (Shatte et al. 2019).

In the present study, the data used for experiments are structured mental‐health monitoring data, not naturally occurring posts on social media or free‐text clinical data. A semantic transformation strategy for structured‐to‐text is applied to enable a clinical language model to be used on these structured records. The patient‐level attributes are presented in semantically meaningful clinical descriptions but retain their original values (Li et al. 2022). These representations are then fed into ClinicalBERT to be trained to understand contextually related relationships between demographic, behavior, clinical, and treatment information. Therefore, ClinicalBERT is not applied to pre‐existing clinical narratives, but rather it is applied to learn contextual representations from semantically transformed structured clinical records.

In recent years, the models and methodologies developed in the field of DL have shown the increasing importance of interpretable hybrid learning strategies for intelligent healthcare systems, which are becoming more prevalent and crucial for intelligent healthcare systems (Khan et al. 2025b). The proposed methodology builds upon this line of research by integrating explainability mechanisms with contextual representation learning to improve the transparency and interpretability of model predictions (Hasib et al. 2023). Recently, XAI techniques have been combined with DL architectures to enhance the interpretability of the models' predictions, without compromising their predictive accuracy (Özüpak et al. 2025). LIME and SHAP can give both local and global interpretations of the model predictions, complementing the analysis of the model factors involved in choosing the prediction (Tang et al. 2021).

This study's main methodological advancement is a semantic transformation framework designed to structure the various attributes of mental health monitoring and transform them into clinically organized sequences of text for ClinicalBERT processing. The framework is not intended to change the inner structure of the transformer but rather to propose a semantic organization strategy that can be reproduced to keep the structured information as it was while providing the possibility of language representation in context (Alpsalaz et al. 2025b; Jiang et al. 2026). The contribution is further explored using component‐wise sensitivity analysis and compared to traditional machine learning (ML) models and the latest tabular transformer models such as SAINT and FT‐Transformer.

While AI‐driven mental health research is steadily growing, there are significant hurdles, such as effectively capturing heterogeneous clinical and behavioral attributes, representing contextual relationships between structured variables, and making interpretable predictions. In this study, we will thus explore the possibility of using a structured‐to‐text semantic representation together with ClinicalBERT to achieve better mental health outcome classification than traditional ML and transformer‐based tabular methods. The proposed framework is compared with Random Forest (RF), XGBoost, Bidirectional Encoder Representations from Transformers (BERT), TabTransformer, Deep Neural Network (DNN), SAINT, and FT‐Transformer models with various classification metrics and other validation analyses. This study has the following significant contributions:

- A structured‐to‐text semantic transformation strategy is designed to transform structured mental health monitoring records into semantically organized clinical representations that can be semantically processed in context by ClinicalBERT.
- A clinical language model based on transformers, called ClinicalBERT, is explored to learn representations from the transformed structured records.
- The proposed ClinicalBERT framework shows an accuracy of 90.2%, which exceeds the performance of all the baseline and comparison models tested under the adopted experimental protocol.
- LIME and SHAP are used to give local and global explanations of ClinicalBERT predictions as complementary.
- To validate the stability and robustness of the framework, it is evaluated through class‐wise performance analysis, five‐fold cross‐validation, confidence interval (CI), statistical significance testing, ablation analysis, and sensitivity analysis.

The remainder of this paper is organized as follows. Section 2 presents the related work and highlights existing gaps. Section 3 describes the proposed methodology, dataset, and preprocessing techniques, including model architecture and explainability techniques, and provides experimental results and evaluation. Section 4 presents results and discussions of applied models and offers a comparative analysis with existing studies. Finally, Section 5 concludes the paper with future research directions.

## 2 Related Work

Studies of mental health monitoring and diagnosis have begun to embrace the incorporation of AI/ML (Du et al. 2021) and DL algorithms to tackle issues related to the early detection, personalization of treatment, and prediction of outcomes (Jin et al. 2026a). Traditional approaches relied largely on clinical surveys, professional judgment, and systems that were not necessarily scalable and applicable to non‐homogeneous populations of patients. As transformer‐based architectures have become available, text analysis models such as BERT and domain‐specific versions have been applied to mental health text analysis, allowing a deeper contextual interpretation of patient stories (Wang et al. 2025). It is the paradigm shift between the old and outdated statistical models, on the one hand, and the new modern AI systems, on the other hand, that can deliver explainable and viable solutions to the process of mental health monitoring that are human‐friendly (Chu et al. 2025). The most recent research on mental health monitoring focuses on multimodal data (text, speech, video, physiological signals) and DL.

Liu et al. (2021) presented an example of an NLP‐based system that uses information on Reddit to monitor the general feeling and behavioral changes in the COVID‐19 pandemic in North Carolina. To identify the most important societal issues, including distancing, disinfection, and mask usage, they gathered posts in 18 location‐specific subreddits (March–August 2020) and used a combination of feature engineering, LDA topic modeling, a custom named‐entity recognition (NER), and BERT‐based sentence clustering. The described multimodal approach to analysis is useful to demonstrate changing community attitudes, with mask acceptance growing as time goes by.

Ahmed et al. (2022) reported that the number of such modeling studies has risen considerably at the height of COVID‐19, indicating a focus on mental health surveillance over the internet. Minimal details are provided, but the emphasis lies on comparing the ML methods, which may involve both classical models (e.g., SVM, RF) and DL methods used on the text data of social media. Although the entire methodology is not available, this article highlights the increasing number of studies that investigate the application of social media in mental health identification during a pandemic. It is likely to discover model performance variants and the growing scale of datasets and can be problematic in all domains, such as data labeling and generalization. But without model or data descriptions, this work can be considered a validation of field‐wide trends, but not a technical landscape. Mansoor and Ansari (2024) developed an AI‐based early crisis detection system that is built on multilingual social media posts. They trained a BERT‐based NLP model on 996,452 Twitter, Reddit, and Facebook posts by adding temporal information through an LSTM with attention. Their deep‐learning multimodal pipeline (NLP + time series) reported an accuracy (F1 = 0.856) of 89.3% for mental health crisis seven days in advance. The merits of this work include its size and cross‐lingualism, yet it also creates ethical and privacy issues (self‐surveillance on social media) and is based on post labels. Ding et al. (2025) compared ML and DL in the process of detecting mental diseases in social media. On a Kaggle dataset of social posts (with several classes), they observed that transformer‐based models (ALBERT) were a little better than the traditional algorithms (RF: ALBERT F1 = 0.965 vs. RF F1 = 0.93); however, they also found that ML algorithms (logistic regression, LightGBM) were competitive with superior interpretability. It turns out to be a trade‐off: DL shows small accuracy gains on moderate‐scale data, but simpler models are fast and transparent. The study based on social media text shows effectiveness but suffers from drawbacks concerning the representativeness of the data and the ability to explain the models (Li et al. 2026).

Non‐social multimodal fusion has been investigated as well. Xing et al. (2024) addressed the problem of EEG+audio depression recognition. They introduced EMO‐GCN, which is a graph neural network that combines 128‐channel EEG data and patient interview audio characteristics to classify major depressive disorder (MDD). EMO‐GCN builds a set of graphs to model the interactions between modalities with 96.30% accuracy on the MODMA dataset. Such high accuracy indicates the strength of graph‐based fusion, although this model is trained on EEG/audio and may not be generalizable to other disorders and data. Sadeghi et al. (2024) reported a large language model and image analysis‐based multimodal depression gr. They obtained the text features of transcripts (LLM‐based) and the facial expression features of video frames on the DAIC dataset of clinical interviews. Their optimal model (text + speech‐quality features) has a PHQ‐8 error of 2.85. It indicates that semantic cues involving depression can be distilled with LLMs, although vision features do not add much value. Its strength is using Transformer NLP, but this is constrained by the small size of the interview sample and non‐naturalistic data. Xu et al. (2025) put voice and text in other fusions and measured depression. They trained pretrained Wav2Vec2.0 on audio and BERT on text and cross‐linked them using a multi‐scale CNN and BiLSTM. Their model was tested on Chinese (CMDC) and English (DAIC) corpora and exceeded uni‐modal baselines (e.g., F1 gain of + 0.0103 on CMDC). Plasticity is shown by cross‐language success, but the model is not complex and has only been tested on benchmark corpora, not in‐the‐wild data. In general, these multimodal fusion studies (Yin et al. 2024) show that using modalities (EEG+audio, text+audio) enhances mental‐health inference, but it is important to pay attention to the fusion strategy and large training data.

In addition to mental health applications, Demirtaş Alpsalaz et al. (2025) state that state‐of‐the‐art transformer‐based DL models have proved useful in the classification of brain‐related diseases. In the context of Alzheimer's disease diagnosis, recent research has demonstrated that the incorporation of vision transformer architectures has the potential to significantly enhance diagnostic accuracy by extracting contextual features and a deep representation of the image. These findings further underscore the versatility of transformer‐based AI systems in clinical decision‐support applications.

Physiological signals have also been subjected to DL. Ksibi et al. (2023) provided a dataset of the resting‐state EEG of a multi‐modal dataset of MODMA processed with CNN. They reported 97% accuracy in MDD vs. controls after 25 epochs of training and observed that adding demographic data (age, gender) would enhance generalization. This demonstrates that EEG biomarkers of depression can be extracted by deep networks. The performance is spectacular, although the model can be overfitting this data, and you have not tested the performance on multi‐site data. The collected wearable heart rate variability (HRV) was evaluated by Jo et al. (2024) as a digital representation of mood. They were able to measure smartwatch PPG‐HRV (SDNN, RMSSD, LF/HF, etc.) and correlate them with PHQ‐9/GAD‐7 scores in a 4‐week study (*N* = 47). They discovered a few important negative relationships (reduced HRV and increased symptoms). This will not be a predictive model, but it implies that mobile sensor data can passively track mood. One weakness is the small sample size and observational nature; however, it will encourage further AI‐based studies of wearable signals.

In some work, the social‐network dimension is present. Mao and Han (2025) employed a Graph Convolutional Network (TextGCN) that employs emotion‐sensitive embeddings to improve the detection of depression on social media text. They also reached much higher accuracy on five Twitter/Reddit datasets by substituting one‐hot inputs with domain‐specific model embeddings (MentalBERT, etc.). This takes advantage of the graphical nature of the word‐document relationships as well as affective content. Their model has greater detection accuracy through the use of emotional context, though it uses pre‐trained specialized models and does not use real‐world social network graphs. Yokotani et al. (2025) explicitly made use of social graph data available in an online game. They built a social network of 142,147 users (friend edges and communication logs) in a Japanese MMO and used GraphSAGE to make predictions of social anxiety disorder. The graph model with 819 labeled users (142 SAD) obtained F1 = 0.717. This is a new approach to mining interpersonal behavior (friends, visits) as biomarkers. It shows that social network features can work in mental health detection but are specific to one platform/population. In short, we also added papers that use and those without social‐network features: the main theme is multimodal DL (text, audio, video, sensors) for mental health and sentiment analysis, and some studies explicitly use social‐network data where possible (Duan et al. 2026).

In other studies, they develop personal or adaptive systems. Song et al. (2024) proposed a macro–micro‐Transformer model of daily mental health monitoring. Their system generates global emotion embeddings with user‐specific layers using information on 298 participants (wearable sensors + speech diaries + self‐reported emotions). A multitask model of emotion prediction combining physiological (HRV) and speech features had a concordance of 0.503. This indicates the personalization of multimodal mood detection, but the overall performance is low (CCC∼0.5). In the same manner, Kim et al. (2023) created app software that detects adolescent depression. Their application gathered passive‐phone data (screen time, distance between locations, and number of calls/text messages) of 24 depressed teenagers and 10 controls. They were able to predict depression diagnosis at a cross‐validated accuracy of about 77% using a deep DNN (with fuzzy feature selection). They also estimated antidepressant response with a high probability (∼76%). Long phone use and call length were important predictors. Real‐world behavioral data and dual prediction are strengths; the small sample and possible overfitting that needs validation in cohorts are weaknesses. In both works, it is demonstrated that continuous mood tracking can be achieved by training DL models on multimodal personal data from mobile and wearable devices, where personalization and data sparsity are still issues.

In recent medical AI research, Alpsalaz et al. (2025a) presented attention‐based hybrid DL architectures which have also shown that they can represent features and achieve accurate diagnosis by employing effective feature fusion mechanisms. Such attention‐driven learning strategies allow the models to learn complex contextual relationships between clinical variables to increase the reliability of predictions, which in turn aids in facilitating intelligent healthcare decision‐support systems. Lastly, a few works apply deep graph neural networks to non‐social networks. Zhao et al. (2025) proposed depression severity in video multi‐instance GNN, DepressionMIGNN. It splits conversations (e.g., the AVEC dataset) into utterances and constructs a graph with temporal edges and R‐GCN and GAT layers to coordinate multimodal features. On AVEC2019, DepressionMIGNN reached CCC = 0.554 (RMSE = 4.61) and had the best performance compared to baselines. It can model depression as a long‐lasting state successfully with the help of graph fusion, but it is very complicated (Wang et al. 2026).

In general, the chosen works cover a variety of modalities and techniques: social media text mining, smartphone sensor analytics, biomedical signals, and all use DL. We added both to the range of possible combinations of all mental health monitoring with DL. Overall, these articles indicate that assessment of mental health using multiple data sources (language, audio, visuals, physiology, and social graphs) with deep models can do a better job, though both methods have trade‐offs in terms of data requirements, generalizability, and interpretability.

## 3 Research Proposed Methodology

The suggested methodology represents a comprehensive approach to mental health monitoring that involves a synthesis of structured clinical information and unstructured patient histories based on the use of ML and DL algorithms. The pipeline will preprocess raw data, extract features, and train deep models to transform raw data into useful representations in a systematic way. The predictive performance of both baseline and proposed models is benchmarked using both evaluation metrics to make sure that the comparison is fair. In addition, explainable AI such as LIME and SHAP are introduced to achieve transparency in the decision‐making process, and this aspect makes the system reliable to be applied in clinics. The proposed methodology framework diagram is shown in Figure 1.

**FIGURE 1:**
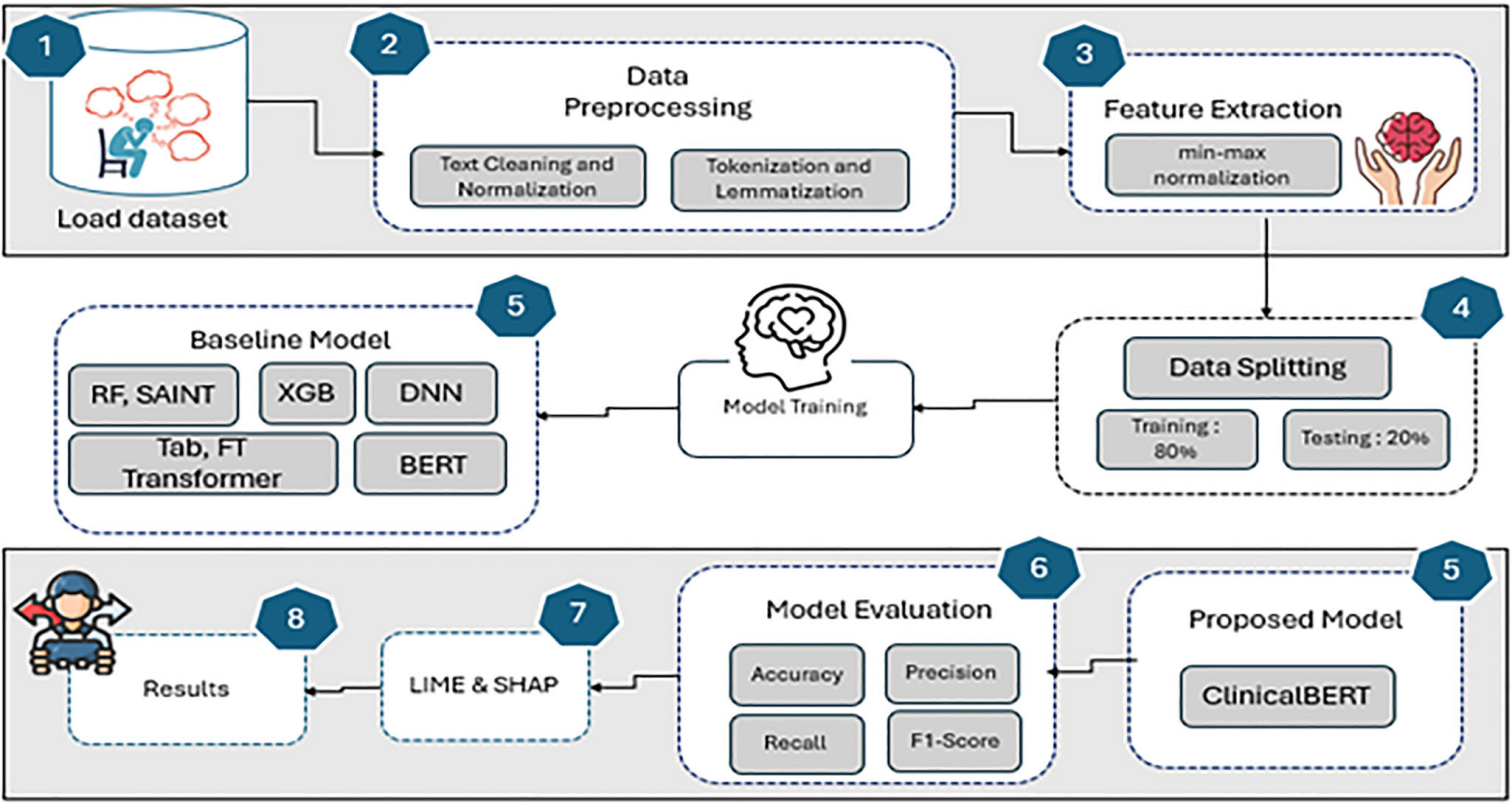
Proposed methodology framework illustrating the complete workflow of the proposed ClinicalBERT‐based mental health monitoring system.

### 3.1 Dataset Description

From the Mental Health Diagnosis and Treatment Monitoring Dataset, which is available in the open Kaggle data repository, the experiments presented in this study were conducted. The dataset was designed as a comprehensible baseline for mental health diagnosis and treatment monitoring by combining information from demographic data, clinical observations, behavior, therapeutic interventions, medication history, and treatment outcomes into comprehensive patient‐level data. It offers a holistic view of several variables that affect mental health conditions and the success of treatment, which makes it applicable to supervised learning and intelligent healthcare analytics. The data is *N* patient records, with each record having *D* variables (numerical and categorical). Numerical variables are the ages of patients, stress level, sleep quality, duration of therapy, and other quantitative health measures; categorical variables are gender, diagnosis, type of therapy, medication(s) prescribed, and outcome of therapy. Let the complete dataset be represented as Equation (1).

(1) $$D={\left\{\left(X_{i},Y_{i}\right)\right\}}_{i=1}^{N}$$

where $X_{i}$ denotes the feature vector corresponding to the ith patient and $Y_{i}$ represents the associated mental health outcome class. The supervised learning objective is therefore defined as Equation (2).

(2) f:X→Y

where f(·) denotes the classification function learned by the proposed framework. The mix of the data set facilitates the proposed models to learn demographic, behavioral, therapeutic, and clinical outcomes at the same time. This multidimensional information can give a detailed picture of the patient's condition and help to identify complex links between stress levels, sleep quality, medication, diagnosis, and therapeutic response. The dataset used is structured clinical monitoring data gathered for research and benchmarking, whereas datasets with raw electronic health records or physician notes would not have been available. It can then be considered as a semi‐structured clinical data set, not just a clinical narrative. To support contextual semantic learning with ClinicalBERT, each structured patient record was automatically converted into a clinical narrative in a standardized format by the proposed structured‐to‐text semantic representation module. This transformation retains all the clinical data and creates meaningful textual sequences for the transformer‐based language models (Cai et al. 2026). An extensive exploratory analysis was conducted before the development of the models to investigate the distribution of features, class balance, data quality, and missing values. Descriptive statistics for numerical variables and frequency distribution for categorical variables were used to analyze the data. The dataset had very few missing values, which were also rectified as part of data preprocessing using suitable numerical data imputation and categorical replacement methods. In addition to this, it was important to ensure consistency of data prior to model training by carefully checking for duplicate records and inconsistent representations of attributes.

The data were then systematically preprocessed through a pipeline of steps, such as handling missing values, eliminating duplicate data, encoding the categorical features, normalizing the numerical features, identifying outliers, structuring the data into text and transforming it into a semantic matrix for the ClinicalBERT model, and partitioning the data into distinct strata for efficient training. This preprocessing framework provided both traditional ML models and transformer‐based models with optimal input representations based on the learning mechanisms of each model. A summary of the characteristics of the Mental Health Diagnosis and Treatment Monitoring dataset is displayed in Table 1.

**TABLE 1:** Summary of the characteristics of the Mental Health Diagnosis and Treatment Monitoring dataset.

Characteristic	Description
Dataset name	Mental Health Diagnosis and Treatment Monitoring dataset
Data source	Kaggle repository
Dataset type	Synthetic clinical mental health monitoring dataset developed for research and educational purposes
Total records (samples)	500 patient records
Total variables	17 variables (16 input features + 1 target variable)
Target variable	Outcome
Outcome classes	Improved, no change, deteriorated
Prediction task	Multi‐class classification
Feature types	Numerical, categorical, temporal, and clinical variables
Numerical features	Age, symptom severity, mood Score, sleep quality, physical activity, treatment duration, stress level, treatment progress, adherence to treatment (%)
Categorical features	Gender, diagnosis, medication, therapy type, AI‐detected emotional state
Temporal feature	Treatment start date
Identifier feature	Patient ID
Validation strategy	Five‐fold cross‐validation

Overall, the adopted dataset is a well‐structured and comprehensive benchmark to test intelligent mental health monitoring systems. The proposed framework allows realization of this integration of demographic, behavioral, clinical, and therapeutic information, paving the way for the investigation of complex patterns in mental health, while simultaneously providing support for traditional ML algorithms and for contextual transformer‐based semantic models (Kalpana et al. 2024).

### 3.2 Preprocessing Data

A very crucial step is preprocessing of data so that one ends up with organized as well as noise‐free data, which can be used to train the model. As the data includes both nominal (e.g., gender, therapy type, medication) and numerical (e.g., stress levels, sleep quality) features, various preprocessing plans are implemented in a systematic manner. This is to ensure better quality of data, less bias, and normalization of values to achieve better model convergence and generalization (Ishfaq et al. 2025). The gaps in the values were filled in with the relevant methods based on the type of feature. For attributes that are numerical, mean imputation was used, computed by Equation (3)

(3) $$x_{ij}^{*}=\left\{\begin{array}{c} x_{ij},\;if\;x_{ij}\neq <NaN\\ \mu_{ij},\;if\;x_{ij}\leq NaN \end{array}\right.\;$$

where xij,μj is the value of the jth feature for the ith sample, and μj is the mean of feature *j*. For categorical features, missing values were replaced with the mode (most frequent category). Normalize numerical features (Ahmad et al. 2023). To be able to compare across features of varying scale, *z*‐score normalization has been used, and computed using Equation (4).

(4) x∼ij=σjxij−μj

where μj are the mean and standard deviation of feature *j*. This transformation guarantees zero mean and unit variance, making models such as DNN and ClinicalBERT converge faster. Embedding vectors were used to encode categorical attributes (gender, diagnosis, and type of therapy) instead of one‐hot encoding because embeddings are semantically similar (Jin et al. 2026b). Let 𝑐 represent a nominal variable with *k* unique categories. Its definition is the embedding representation computed by Equation (5).

(5) $$E\;\left(c\right)=W\cdot one_{h}ot\left(c\right)$$

where $W\in Rd\times kW\in R^{d\times k}W\in Rd\times k$ is the embedding matrix, and *d* is the embedding dimension. This approach is particularly effective for models like TabTransformer and DNN.

### 3.3 Train–Test Split

Stratified sampling was used to split the dataset into training and testing sets to maintain the distribution of classes. Formally computed by Equations (6)–(8).

(6) $$D=D_{\mathrm{train}}\cup D_{\mathrm{test}},D_{\mathrm{train}}\cap D_{\mathrm{test}}=\emptyset$$

(7) $$D=D_{\mathrm{train}}\;\cup D_{\mathrm{test}},\;D_{\mathrm{train}}\cap D_{\mathrm{test}}$$

(8) $$D=D_{\mathrm{train}}\cup D_{\mathrm{test}},D_{\mathrm{train}}\cap D_{\mathrm{test}}=\emptyset$$

With $|D_{\mathrm{train}}|=\alpha |D|,|D_{\mathrm{test}}|=\left(1-\alpha \right)|D||D_{\mathrm{train}}\left|=\alpha |D|,\;\right|D_{\mathrm{test}}|=\left(1-\alpha \right)|D||D_{\mathrm{train}}|=\alpha |D|,|D_{\mathrm{test}}|=\left(1-\alpha \right)|D|$, where (80% training, 20% testing).

### 3.4 Feature Extraction

The next important process in converting the processed dataset into a form that can be useful to ML and DL models is feature extraction. In this work, both structured (tabular) and unstructured (textual) data provided features and allowed viewing of patient records holistically. In this way, the models will be able to address both numerical patterns of demographic and clinical characteristics (Kasanneni et al. 2025) and the semantic meaning of textual notes computed by Equation (9).

(9) $$E\;\left(x\right)=W\cdot O\left(x\right)$$

where *O*(*x*) is the one‐hot encoded vector of the categorical feature $W\in Rk\times dW\in R^{k\times d}W\in Rk\times d$, which is the embedding matrix mapping categories to a dense *d*‐dimensional space, and *E*(*x*) is the resulting dense feature vector used for learning. In the case of structured data, statistical normalization procedures were used to scale the numerical variables age, stress levels, and therapy sessions. Categorical attributes (e.g., gender, type of medication) that were coded as the dense numeric vectors (e.g., gender, type of medication) were one‐hot encoded. Given a categorical feature *x* with *k* distinct categories, the embedding transformation is computed by Equation (10).

(10) S=n1i=1∑nhi

where hi represents the hidden state embedding of the ith token, *n* is the total number of tokens in the sequence, and *S* is the aggregated sentence embedding representing the entire clinical note. In textual data, transformer‐based model embeddings (e.g., BERT and ClinicalBERT) were used to encode semantic connections (Li et al. 2027). All words in every sentence or clinical note were tokenized, and a contextual embedding vector was created for each token. The last sentence embedding was acquired by the average pooling of all contextual token vectors computed by Equation (11).

(11) $$F=\left[F\mathrm{struct};F\mathrm{text}\right]\;$$

Fstruct represents the embedding from textual data F is the combined multi‐modal feature representation. Beyond that, to trade off predictive power versus interpretability, hybrid feature representations were adopted. This implies that statistical data (e.g., stress levels, type of therapy) were combined with semantic subtexts of clinical notes into a single input vector (Kim et al. 2021).

### 3.5 Structured‐to‐Text Semantic Representation for ClinicalBERT

The dataset used is made up of structured attributes like demographics, diagnosis, treatment, and behavior, but transformer‐based language models like ClinicalBERT need natural language sequences as input. To fill this gap, a structured‐to‐text semantic transformation module was added to the proposed framework. All records were automatically transformed into a standard clinical narrative, and a coherent textual representation of the patient record was generated for each patient in a way that retained the same meaning as the original record while capturing the semantic relationships between all the attributes that appeared in the original record: demographic information, diagnosis, treatment history, medications, stress level, sleep quality, and clinical outcome. This transformation allowed each patient record to be encoded as a clinical document in its context, instead of as a set of variables in a table. A patient record with structured attributes like age, gender, diagnosis, therapy type, medications, stress level, sleep quality, and outcome of treatment, for instance, was translated into a standardized narrative in the following format.

The treatment outcome for a 32‐year‐old female patient with generalized anxiety disorder who received cognitive behavioral therapy and selective serotonin reuptake inhibitor medication recorded a high level of stress and poor sleep quality with partial clinical improvement. Such automatically generated narratives maintain clinical context and semantic relationships between various patient features, allowing ClinicalBERT to learn contextual representations that cannot be directly captured from structured numerical features. The bidirectional attention mechanism enables ClinicalBERT to effectively leverage these relationships as part of the learning process, which is not possible with traditional tabular approaches (Ye et al. 2024). As a result, each patient record is encoded as a high‐dimensional contextual embedding that models both local and global semantic interactions, enhancing the model's ability to capture intricate patterns of mental health. The process of changing word meanings is formally computed as Equation (12).

(12) $$T_{i}=G\left(X_{i}\right)$$

where $X_{i}$ denotes the structured patient record, G(·) represents the semantic text generation function, and $T_{i}$ is the generated clinical narrative supplied to ClinicalBERT. The generated narrative is subsequently tokenized using the ClinicalBERT tokenizer that is computed with the help of Equation (13).

(13) $$S_{i}=\left\{w_{1},w_{2},\text{…},w_{n}\right\}\;$$

where each token sequence is encoded into contextual embeddings by the transformer encoder that is computed with the help of Equation (14).

(14) $$H=\;\mathrm{ClinicalBERT}\left(S_{i}\right)$$

The semantic representation of the overall patient record is obtained by the contextual embedding of the [CLS] token that is computed by Equation (15).

(15) $$z_{i}=H_{\mathrm{CLS}}\;$$

The result of which is fed into the classification layer, where it is used to predict mental health outcomes. This structured‐to‐text semantic transformation ensures that structured healthcare record information is connected to transformer‐based contextual learning while maintaining all the clinical information within each patient record. To enable the clinical organization of the textual representation of heterogeneous mental‐health monitoring attributes, the proposed structured‐to‐text semantic transformation was developed to transform the mental‐health monitoring attributes prior to contextual encoding by ClinicalBERT. The transformation treats each of the structured attributes as a single numerical or categorical variable; in the transformation, related variables are grouped by their semantic relationship, such as demographic data, clinical diagnosis, severity of symptoms, behavioral data, treatment data, and monitoring data. Each attribute is transformed into a natural‐language attribute‐value pair, and related attributes are organized in a consistent context. The resulting representation retains the original information content while also being a “semantic sequence” that is ordered and can be processed by the ClinicalBERT tokenizer and contextual encoder. So, the methodology is not about changing the internal architecture of ClinicalBERT itself; instead, it is about the routinely repeatable semantic transformation and contextual encoding process, which allows the structured attributes of mental‐health monitoring to be represented as clinically structured textual sequences.

### 3.6 Applied Models

The dataset was evaluated using various ML and DL models after a feature extraction. These models have been chosen to compare traditional methods and the latest methods in clinical outcome prediction. This was driven by the desire to compare standard baseline algorithms with advanced neural architectures to identify which models are more accurate, robust, and clinically relevant (Hinduja et al. 2022).

#### 3.6.1 Baseline Models

Four baseline models were used to provide a reference performance of mental health prediction: RF, XGB, BERT, and TabTransformer. RF and XGB are tree‐based ensemble ML techniques that use multiple decision trees to enhance predictive accuracy and decrease overfitting. RF employs bagging and majority voting, but XGB uses gradient boosting and optimized regularization and is resistant to complexities in tabular and numerical data.

BERT is a transformer‐based language model that considers contextual semantic information of textual characteristics, allowing the analysis of unstructured data. TabTransformer builds upon the transformer architecture into the tabular data format and can learn interaction between categorical and numerical features (Naz and Khan 2026). The DNN is a multi‐layer feedforward artificial neural network that learns complex nonlinear relationships from high‐dimensional data using multiple hidden layers. This study employs an input layer, a series of fully connected hidden layers with ReLU activation, dropout regularization to prevent overfitting, and a Softmax output layer for multi‐class mental health classification. With the goal of effectively capturing complex interactions between features within the mental health monitoring dataset, the network was trained with the Adam optimizer and cross‐entropy loss. It is also known for its hierarchical feature learning ability, which allows it to give more accurate predictions than traditional ML models and automatic high‐level representations of the input data. SAINT is a transformer‐based architecture for tabular data, which uses attention mechanisms to learn relationships between features and instances of data. FT‐Transformer represents each feature as a token and learns feature interactions using self‐attention (Zhuang et al. 2024). Algorithm 1

ALGORITHM 1 **Structured‐to‐Text Semantic Transformation and ClinicalBERT Prediction**

**Table:** 

**Input**: Structured patient record R Attribute groups G ClinicalBERT tokenizer T Pretrained ClinicalBERT model B **Output**: Predicted mental‐health outcome $Y_{hat}$ 1. Load structured patient record R. 2. Handle missing values and normalize/encode the required attributes. 3. Divide the attributes into semantic groups: demographic, clinical, behavioral/monitoring, treatment, outcome‐related attributes. 4. For each semantic group: a. Select its corresponding attributes. b. Convert each attribute‐value pair into a textual expression. c. Preserve the predefined semantic ordering. 5. Concatenate the generated expressions into a structured clinical textual sequence S. 6. Apply the ClinicalBERT tokenizer to *S*. 7. Truncate/pad the token sequence to the predefined maximum length. 8. Pass the tokenized sequence through ClinicalBERT. 9. Extract the contextual representation from the encoder. 10. Pass the representation through the classification layer. 11. Calculate the predicted class using the Softmax function. 12. Return the predicted mental‐health outcome $Y_{hat}$.

#### 3.6.2 Proposed Model

ClinicalBERT is a transformer‐based language model that builds upon BERT and is trained on clinical notes and healthcare‐related text. Such specialization allows the model to learn domain‐specific semantics and medical terminology, as well as the contextual constraints of patient data that general‐purpose models (such as vanilla BERT) might ignore. Applied to mental health surveillance, ClinicalBERT helps gain more insight into the ways clinical narratives (Wang et al. 2024), patient records, and treatment notes can reflect the state of psychological conditions computed by Equation (16).

(16) $$H=TransformerEncoder\left(X\right)$$

where *x* is the sequence of token embeddings, and *h* represents the contextualized hidden states produced for each token. In its simplest form (Al Banna et al. 2023), the basic working principle of ClinicalBERT can be mathematically explained as in Figure 2.

**FIGURE 2:**
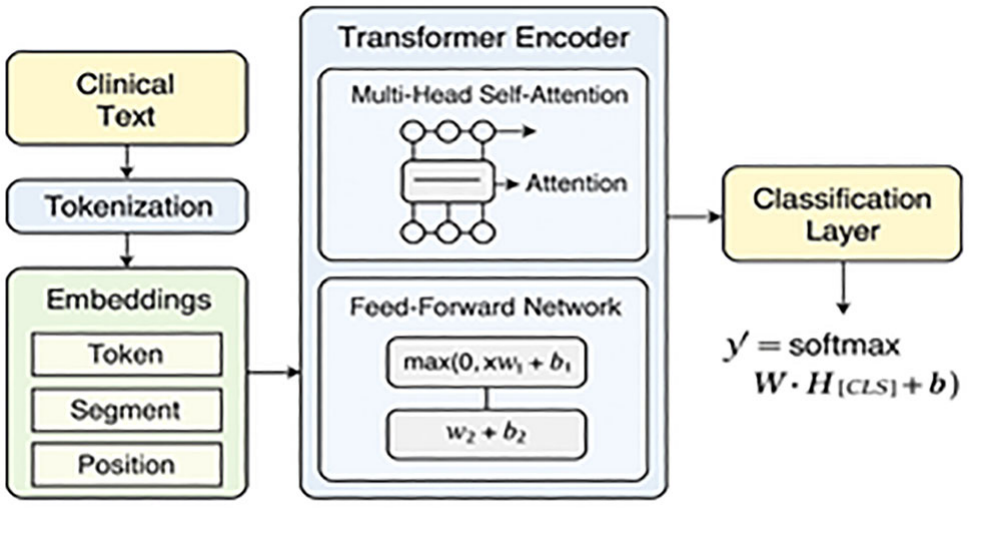
Workflow of the ClinicalBERT model illustrating structured‐to‐text semantic transformation, tokenizer‐based input encoding, contextual feature extraction through bidirectional transformers, and final mental health outcome classification.

#### 3.6.3 Input Representation

First, WordPiece tokenization is applied to clinical text. The tokens are then mapped into a vector embedding space computed by Equation (17).

(17) $$E=E_{token}+E_{segment}+E_{position}$$

The input representation is the totality of token representations, segment representations, and positional representations. This makes sure that not only the word meaning is captured in the model, but also the sentence structure and the position of the token.

#### 3.6.4 Multi‐Head Self‐Attention

The ClinicalBERT model uses self‐attention to learn the relationships between all tokens in a sequence, regardless of their distance, from the model.

- The attention heads each compute scaled dot‐product attention.
- The model uses multiple attention heads simultaneously so that it can learn various semantic relations.

#### 3.6.5 Feed‐Forward Network (FFN)

Every transformer block has a position‐wise feed‐forward network (FFN) that acts upon all the token representations computed by Equation (18).

(18) $$\mathrm{FFN}\;\left(x\right)=\max\left(0,xW1+b1\right)W2+b2$$

This introduces nonlinearity and increases the representational ability of the model.

#### 3.6.6 Residual Connections, Layer Normalization

Other connections hold gradients and improve training. Layer normalization: the activations are centered/scaled, which improves convergence.

#### 3.6.7 Output Representation

Use the [CLS] token embedding as the synthesized representation of the whole sequence. The vector is then forwarded to a classification layer (a softmax or sigmoid) where the mental health outcomes are predicted, computed by Equation (19).

(19) $$y=\mathrm{softmax}\left(W\cdot H\left[\mathrm{CLS}\right]+b\right)$$

### 3.7 Evaluation Measures

To critically assess the performance of the two baseline and proposed models, a few standard metrics were used. The measures provide the quantitative predictive ability of the models in accuracy, precision, recall, and F1‐score, thus providing a complete evaluation of the classification performance. Moreover, the capability of models to differentiate between classes at different thresholds was considered based on the receiver operating characteristic (ROC)‐area under the curve (AUC) score (Shah et al. 2024).

#### 3.7.1 Accuracy

Accuracy is used to measure the number of correctly classified instances out of the total number of samples. It is defined as Equation (20).

(20) $$\mathrm{Accuracy}\;=\frac{TP+TN}{TP+TN+FP+FN}\;$$

where *TP* = true positives, *TN* = true negatives, *FP* = false positives, and *FN* = false negatives.

#### 3.7.2 Precision

Precision shows the percentage of the cases correctly predicted as positive out of all cases that are predicted as positive. This particularly applies in the healthcare environment to reduce false positives. It is defined as Equation (21).

(21) $$\mathrm{Precision}\;=\frac{TP}{TP+FP}\;\;$$

#### 3.7.3 Recall (Sensitivity)

Recall is a measure of the ratio of correctly identified positive cases out of the actual positive cases. When using mental health monitoring, high recall means that actual cases of patients at risk are not overlooked. It is defined as Equation (22).

(22) $$\mathrm{Recall}\;=\frac{TP}{TP+FN}\;$$

#### 3.7.4 F1‐Score

F1‐score is the harmonic mean of precision and recall, which weighs both together to give a single measure of model efficacy. It is defined as Equation (23).

(23) $$F1-\mathrm{Score}\;=\frac{2\cdot Precision\cdot Recall}{Precision+Recall}\;$$

#### 3.7.5 ROC and AUC

The ROC curve compares the sensitivity (recall) versus specificity at various thresholds. The sum of this performance is the AUC. It is defined as Equation (24).

(24) $$\mathrm{AUC}\;=\int_{0}^{1}TPR\left(FPR^{-1}\left(x\right)\right)\;\;\mathrm{dx}$$

where $TPR=\frac{TP}{TP+FN}$ (true positive rate), $FPR=\frac{FP}{FP+TN}$ (false positive rate).

### 3.8 Comparative Input Representation Strategy

An additional comparative experiment was conducted with various input representation strategies to identify whether the proposed structured‐to‐text semantic transformation is responsible for performance improvement or if it is due to simply converting tabular attributes into text. The simplicity of the key‐value textual serialization allowed them to first evaluate ClinicalBERT, where each structured attribute was mapped directly to an attribute‐value pair. Second, ClinicalBERT was tested with the suggested structured‐to‐text semantic transformation task, where the set of patient attributes was transformed into a coherent clinical representation, but their values remained unchanged. Moreover, both SAINT and FT‐Transformer were directly evaluated with the original structured attributes. Each configuration was assessed using an identical protocol and performance measures to allow a fair comparison.

### 3.9 Experimental Protocol and Model Configuration

A common experimental protocol was used for all baseline and proposed models to facilitate the reproducibility, fairness, and robustness of the experimental evaluation. To prepare the dataset for the ClinicalBERT framework, the missing data were first imputed, the categorical features were encoded into text, the numerical features were normalized, and the structured data were transformed into text through semantic transformation. The dataset was split into 80% training data and 20% testing data via stratified sampling with a fixed random seed of 42 to maintain the original class distribution and results from the experiments. To ensure a fair comparison across all models analyzed, all DL‐based models and transformer models (where applicable) were trained for 20 epochs using the same training conditions when developing the model. When developing the models, all models based on DL and transformer models were trained for 20 epochs using the same training conditions, where applicable, for a fair comparison between the analyzed approaches. Moreover, to test the robustness and generalization ability of each model, a five‐fold cross‐validation method was used, and the accuracy, precision, recall, and F1‐score were chosen as the main evaluation metrics. To further validate the reliability of the proposed ClinicalBERT framework, additional analyses such as CI, statistical significance testing, ablation experiments, and sensitivity analysis were performed.

To obtain the contextually learned semantic representations, the proposed ClinicalBERT framework used the publicly available Bio_ClinicalBERT pretrained transformer model and its associated tokenizer to process the clinical narratives generated. The model was optimized with the AdamW optimizer using a learning rate of 2 × 10^−^^5^, a maximum sequence length of 128 tokens, and a batch size of 16. An AdamW optimizer was used with a learning rate of 2 × 10^−^^5^, while a maximum sequence length of 128 and a batch size of 16 were adopted to balance the computational efficiency and contextual representation capability of the model. To avoid overfitting, a dropout rate of 0.3 was added to the model, and the cross‐entropy loss function was used during optimization. The rest of the baseline and DL models were chosen and used with the most frequently adopted optimization strategies with the hyperparameters that are recommended for each model, using the same number of epochs to train each model to give an unbiased comparison. Python, PyTorch, Scikit‐learn, XGBoost, and the Hugging Face Transformers library were used to implement all experiments in the Google Colab environment with the help of an NVIDIA Tesla T4 GPU. Hyperparameter configuration settings of all baseline and proposed models are discussed in Table 2.

**TABLE 2:** Hyperparameter configuration settings of all baseline and proposed models.

Model	Model type	Hyperparameter configuration
Random forest (RF)	Machine learning	Number of trees = 200, Maximum depth = None, Minimum samples split = 2, Criterion = Gini, Random seed = 42
Extreme gradient boosting (XGBoost)	Machine learning	Number of estimators = 200, Maximum depth = 6, Learning rate = 0.1, Subsample = 0.8, Random seed = 42
BERT	Transformer	Pretrained BERT‐base, Batch size = 16, Max sequence length = 128, Learning rate = 2 × 10^−^^5^, Epochs = 20, AdamW optimizer
TabTransformer	Transformer	Embedding dimension = 32, Attention heads = 8, Transformer layers = 4, Batch size = 16, Learning rate = 2 × 10^−^^5^, Epochs = 20
Deep neural network (DNN)	Deep learning	Hidden layers = (256, 128, 64), ReLU activation, Dropout = 0.30, Batch size = 16, Adam optimizer, Learning rate = 1 × 10^−^^3^, Epochs = 20
SAINT	Tabular transformer	Embedding dimension = 32, Attention heads = 8, Transformer layers = 4, Batch size = 16, Learning rate = 2 × 10^−^^5^, Optimizer = AdamW, Dropout = 0.30, Epochs = 20, Random seed = 42
FT‐transformer	Tabular transformer	Embedding dimension = 32, Attention heads = 8, Transformer layers = 4, Batch size = 16, Learning rate = 2 × 10^−^^5^, Optimizer = AdamW, Dropout = 0.30, Epochs = 20, Random seed = 42
ClinicalBERT (proposed)	Transformer	Bio_ClinicalBERT, ClinicalBERT Tokenizer, Max sequence length = 128, Batch size = 16, Learning rate = 2 × 10^−^^5^, AdamW optimizer, Dropout = 0.30, Epochs = 20, Cross‐entropy loss

### 3.10 Confidence Interval Estimation

A bootstrapping resampling procedure was used to estimate the 95% CI for the reported classification accuracy, which measured the uncertainty in the classification accuracy. Data for evaluation were subsampled with replacement, and the accuracy of the model was computed for each of the subsamples. The median, together with the lower and upper bounds of the resulting bootstrap distribution, was reported as a 95% CI. This approach gives a sense of the range of uncertainty around the observed predictive performance instead of considering the reported accuracy as a single number.

The correct 95% CI equation for the mean accuracy across (*k* = 5) folds is Equation (25).

(25) $$\begin{array}{c} CI_{95\%}=\bar{x}\;\pm t_{0.975,\;k-1}\frac{s}{\sqrt{k}} \end{array}$$

where $\bar{x}$ is the mean classification accuracy across the (*k* = 5) folds, *s* is the standard deviation of accuracy across the five folds, *k* is the number of folds (*k* = 5), ($t_{0.975,k-1})$ is the critical value of the Student's (*t*) distribution with (*k*‐1 = 4) degrees of freedom, and $CI_{95}$ is the 95% confidence interval for the mean accuracy.

### 3.11 Model Explainability: LIME and SHAP

Along with predictive accuracy, explainability is a key factor in the adoption of AI systems in healthcare and mental health monitoring. To build trust and transparency and reduce ethical risks, clinicians and decision‐makers need to know why a model makes certain predictions. This is why LIME and SHAP were used. With these methods, we can visualize the impact of specific characteristics on a specific prediction, which can result in visible and accountable AI through LIME and SHAP. Clinicians will be able to legitimize the decision made by the model according to medical logic (e.g., high stress levels, low quality of sleep, type of therapy). This not only generates confidence but also helps to identify potential biases in the data. Furthermore, the combination of DNN and ClinicalBERT with explainability techniques avoids the problem of advanced models being non‐interpretable, a critical precondition of ethical application in healthcare.

#### 3.11.1 LIME

LIME estimates a complicated model on a local scale by training a less complicated interpretable model on a local prediction. For a given instance x, LIME modulates the input features to produce new samples and measures model prediction changes. It then estimates a weighted linear model to represent the local decision boundary. Although the default LIME formulation optimizes the loss between the black‐box model and a solvable surrogate, a more generalized weighted optimization can be formulated as computed by Equation (26).

(26) $$\hat{g}=\arg\min_{g\in G}g\;\sum_{z^{\prime }\in Z}\pi_{x}\left(z^{\prime }\right)\cdot {\left(f\left(z^{\prime }\right)-g\left(z^{\prime }\right)\right)}^{2}+\Omega \left(g\right)$$

where *z*′ is a perturbed sample of the original instance x $f(z^{\prime })$ is the prediction of the black‐box model on z′. *g*(*z*) is the prediction of the interpretable model, and $\pi_{x}\left(z^{\prime }\right)$ is the locality weighting kernel, often defined as an exponential kernel computed by (27).

(27) $$\pi_{x}\;\left(z^{\prime }\right)=\exp\left(-\frac{D{\left(x,z^{\prime }\right)}^{2}}{\sigma^{2}}\right)\;$$

with *D*(*x*,*z*′) being a distance metric (e.g., cosine or Euclidean) and *σ* controlling the kernel width.

#### 3.11.2 SHAP

SHAP relies on cooperative game theory, and each feature is regarded as a player involved in the prediction. The prediction is distributed fairly by the contribution of each feature with the use of Shapley values. SHAP offers both global interpretability (which measures the overall impact) and local interpretability (which measures which specific prediction was affected). Such duality is indispensable in the field of mental health, as the information about the patient must be appreciated alongside the predisposition of the observed decision in a global situation. The classical Shapley value is under the assumption of independence of features. But in clinical data, such variables as sleep quality and stress level are related. In the case of correlated features, conditional SHAP values are better. SHAP can also be represented as an additive feature attribution model as Equation (28).

(28) $$f\left(x\right)=\phi_{0}+\sum_{i=1}^{M}\phi_{i}x_{i}$$

where $\phi_{0}$ is the expected model output (baseline prediction), ϕ is the contribution of feature *i*, and *M* is the total number of features.

### 3.12 Statistical Significance and Stability Analysis

The performance variability using stratified five‐fold cross‐validation was used to check for the robustness and stability of the proposed framework. Mean and standard deviation were calculated for each of the evaluation metrics over all the validation folds. The average performance of each model was obtained by taking the mean of the values that are computed with the help of Equation (29).

(29) $$\bar{M}=\frac{1}{K}\sum_{k\;=\;1}^{K}M_{k}$$

where $M_{k}$ denotes the evaluation metric obtained during the kth fold and K=5. The corresponding standard deviation was computed as in Equation (30).

(30) $$\sigma =\sqrt{\frac{1}{K-1}\sum_{k=1}^{K}{\left(M_{k}-\bar{M}\right)}^{2}}$$

where σ quantifies the variability of the performance across validation folds. Smaller values of standard deviation signify more stable model behavior and consistent generalization for various partitions of the data set. Both the proposed ClinicalBERT and DNN models demonstrated not only better predictive ability but also greater robustness with lower variability than baseline approaches, on average.

To determine whether the performance differences between the proposed ClinicalBERT model and the baseline models were statistically significant, accuracy scores obtained from five‐fold cross‐validation were compared using paired statistical tests. The scores from corresponding folds were treated as paired observations, allowing a consistent assessment of performance differences between the proposed model and each baseline. To check if the performance difference among the proposed ClinicalBERT model and comparison models was statistically significant. The five‐fold cross‐validation accuracy scores from the same folds were assumed to be paired and independently evaluated with a paired statistical test when comparing ClinicalBERT with each baseline. A significance level of 0.05 was used for statistical significance, and a *p*‐value below 0.05 was considered statistically significant to have a performance difference. This method was employed to separate consistent fold‐level changes from fold‐level differences.

## 4 Results and Discussion

The research results provide a comparative analysis of ML and DL techniques in clinical outcome prediction in detail. The models were all evaluated based on accuracy, precision, recall, and F1‐score so that sensitivity and specificity could be evaluated equally. All of the measures mentioned above indicate the quality of model generalization to various subgroups of patients with minimal false alarms or false misses. The findings may indicate that there are certain noticeable variations among the customary ensemble educators, transformation frameworks, and concrete clinical structures that may exhibit the importance of model choice in relation to clinical uses. The analysis of both the proposed and the baseline models is given in the following sections.

This plot shows the correlation values between various features in the dataset, and it indicates how they are statistically dependent. Strong positive correlations show that the variables tend to rise, whereas negative correlations demonstrate the opposite. On examining these correlations, we can detect overlapping or extremely correlated features that can influence the training of the model. This type of analysis is important to avoid multicollinearity that may bias the performance of the model. The most influential features used to predict mental health outcomes are also shown in the correlation map. This offers helpful hints on how important features are prior to using ML and DL models shown in Figure 3. This visualization shows that stress levels differ according to the age of participants and whether participants are male or female. It shows us trends in which age groups, like younger adults, can be experiencing a higher stress burden compared to the older generation. The differences in stress levels between males and females are also observable in differences in the reaction of males and females to the same stressor. These distributions reflect psychological demographic susceptibility. These lessons can be critical in customizing mental health monitoring systems to individual‐level interventions, as shown in Figure 4.

**FIGURE 3:**
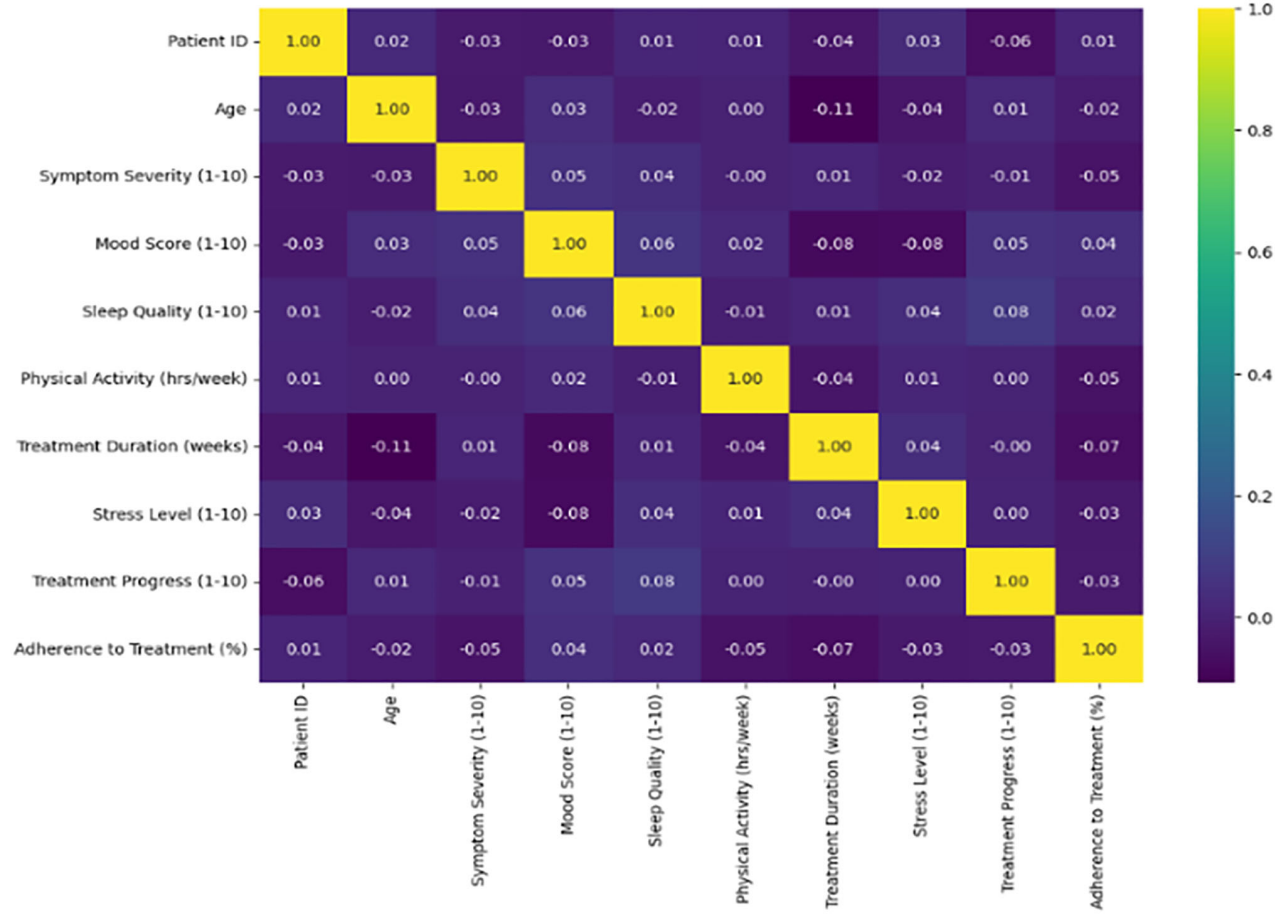
Correlation heatmap showing the relationships between key features in the mental health dataset.

**FIGURE 4:**
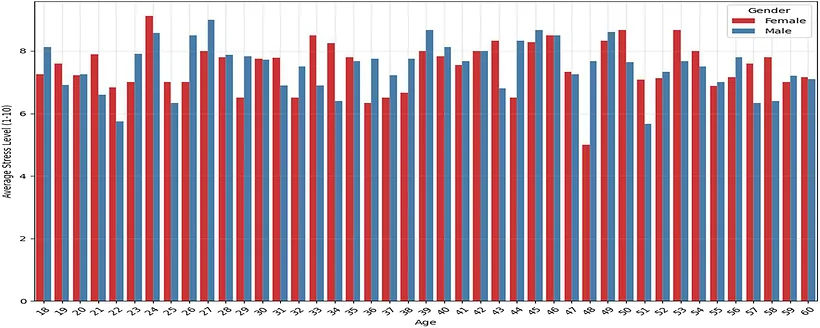
Distribution of stress levels across different age groups categorized by gender, illustrating demographic variations in average stress levels among patients in the mental health monitoring dataset.

This plot presents the mental health outcomes distribution of the patients in the dataset. The results can include positive findings, no findings, and recovered cases. The obvious imbalance between results indicates issues like the distortion of some groups, which should be considered when training the model. The visualization gives a summary of the distribution of prevalence of various conditions in the population. This distribution is important to understand when creating predictive models that are not overfitted to majority classes, as shown in Figure 5.

**FIGURE 5:**
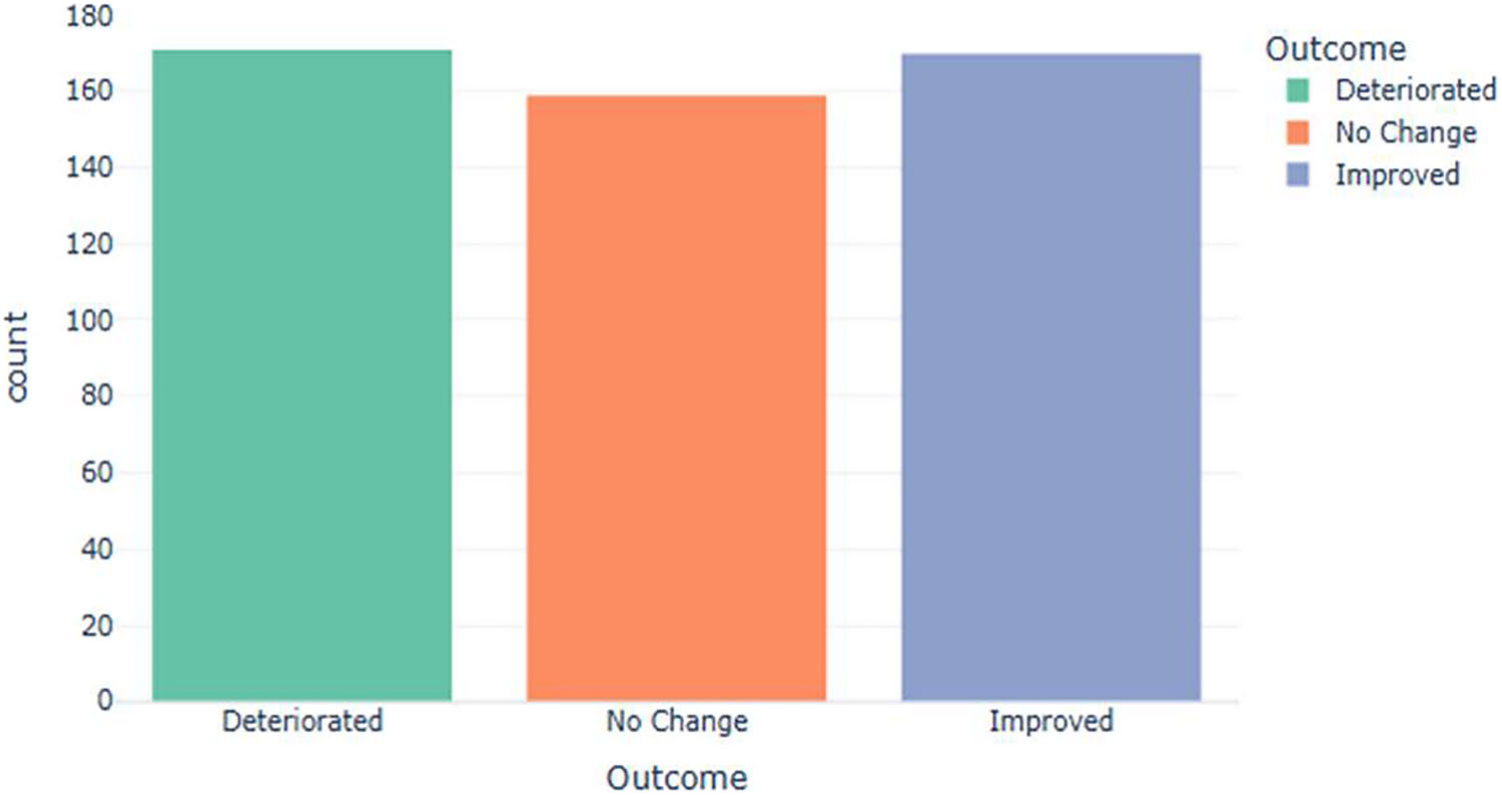
Distribution of patient mental health outcomes across the dataset, showing the frequency of the improved, no change, and deteriorated outcome categories used for multi‐class classification.

The swarm plot gives a descriptive distribution of the number of patients with each category of diagnosis and treatment outcome. A point is a single patient, and it provides a granular perspective of the variation of outcomes in diagnostic groups. Variability and density of groups are also presented using this view over aggregated bar charts. It also shows the overlapping results among various diagnoses, which can be an indication of complex co‐occurring disorders. Such a degree of distribution assists in becoming aware of the level of patient variations that are central to personalized care, as shown in Figure 6.

**FIGURE 6:**
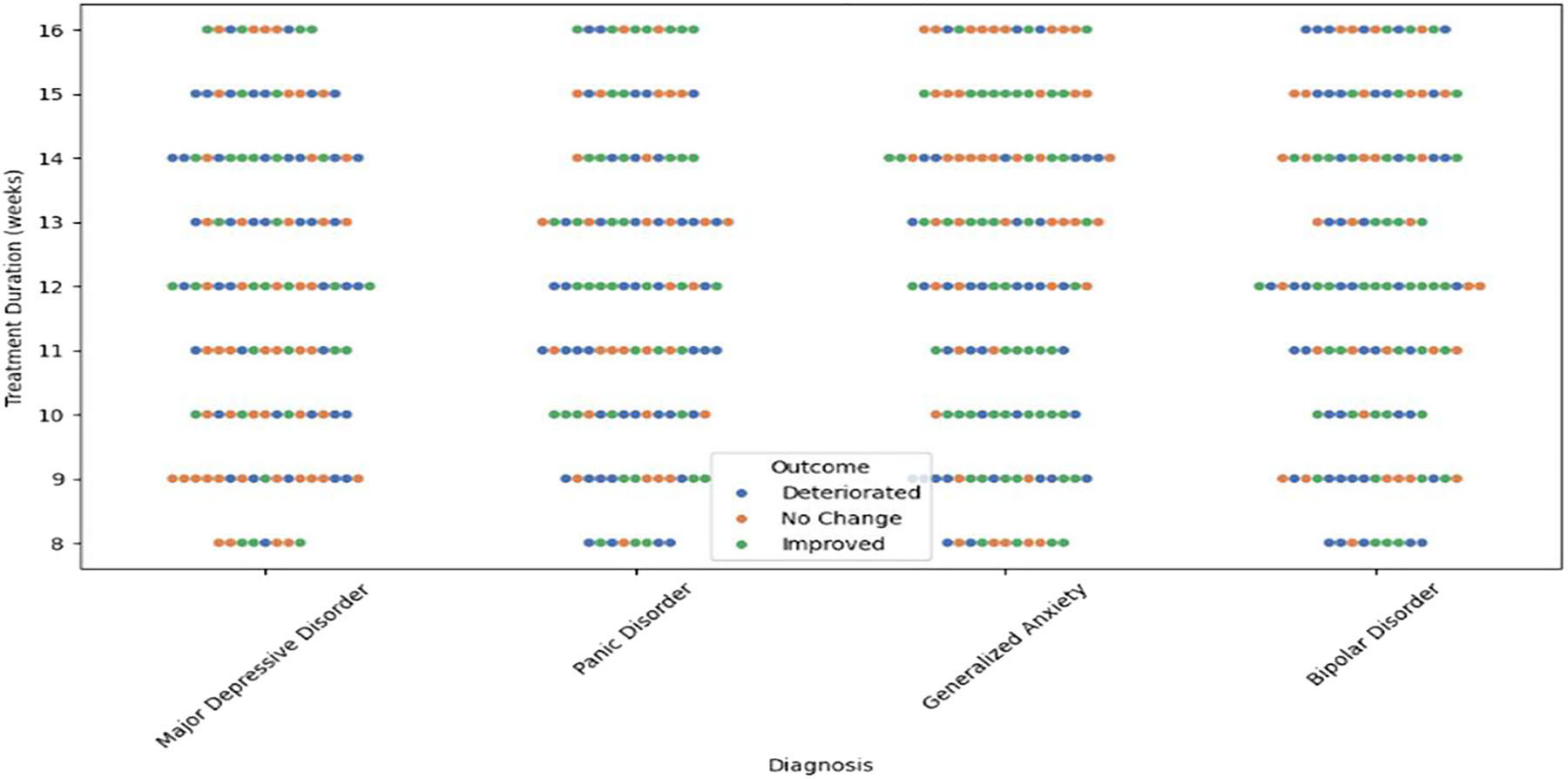
Swarm plot shows patient‐level distribution by diagnosis and mental health outcome.

This figure shows the average sleep quality of patients by type of therapy and medicine. It makes it possible to compare the effects of various treatment methods and medications prescribed to patients on their sleeping patterns. The findings indicate the difference in the average quality of sleep, indicating that certain combinations of therapies and medications have more beneficial effects than others. This kind of grouping can aid in locating the most effective treatment approaches to better sleep, which frequently contributes much to general mental health. In addition, it offers clinicians evidence‐based knowledge on how to personalize interventions. This discussion shows why a combination of treatment methods can be used to improve wellness, as shown in Figure 7.

**FIGURE 7:**
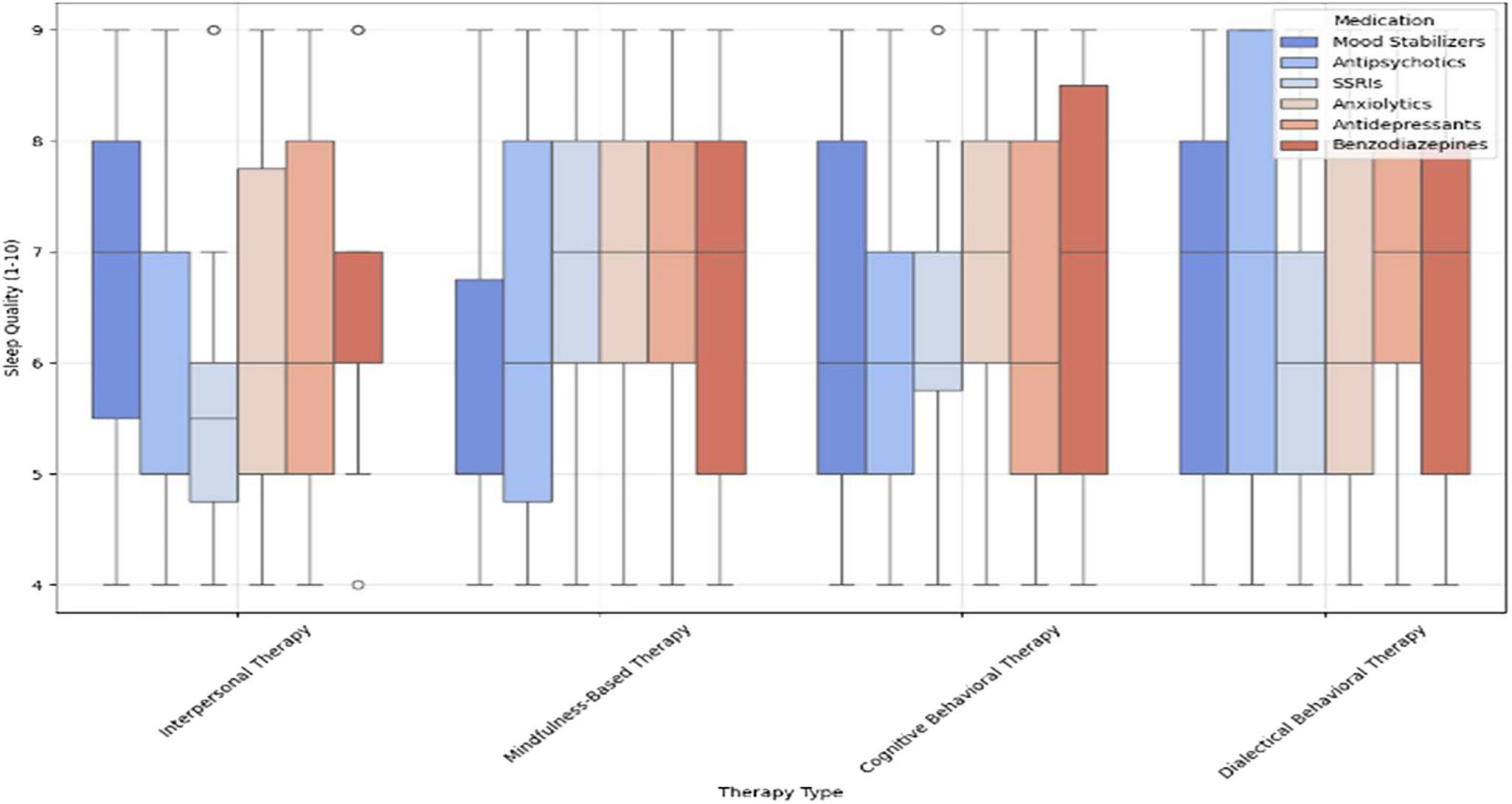
Mean sleep quality values grouped by therapy type and medication, showing the impact of different treatment combinations on patient outcomes.

### 4.1 Baseline Models Results

The RF model had a 78.4% accuracy, 79.2% precision, 78.4% recall, and a 79% F1‐score. The implication of this observation is that the RF could provide reasonably balanced performance and be robust to noisy factors, which could be a significant benefit when it comes to working with mixed‐patient demographic clinical data. But its weak ability to model sequential dependencies or richer interactions of features becomes a bottleneck. The fact that the recall is less than the precision means that RF is prone to false negatives regarding true cases and prioritizes low sensitivity over accuracy. As an interpretable baseline, it is effective, but it does not have the flexibility needed to make more precise medical predictions. XGBoost was a little better than RF with 80.6% accuracy, 79.6% precision, 79.1% recall, and an 80% F1‐score. The enhancement illustrates the effect of strengthening decision trees through sequential corrections of earlier error misclassifications. XGB is more accurate than RF because it captures nonlinear interactions and reduces overfitting better than RF. However, the improvement margin is quite minimal, which suggests that despite the stabilization caused by the boosting, it still struggles with multifaceted or contextual components of healthcare stories. The close balance of accuracy and recall demonstrates that XGB has fewer trade‐offs between false positives and false negatives, but its performance is limited. The initial BERT model reached 82.1% accuracy, a precision of 83.2%, a recall of 81.4%, and an F1‐score of 82.3%. The findings demonstrate a remarkable change compared to the conventional baselines, as the transformer architecture enables BERT to successfully capture contextual relations in clinical text. The fact that its accuracy is greater than recall indicates that BERT is more conservative, aiming to reduce the number of false alarms and, in some cases, false negatives. Such a trade‐off is tolerable in some medical situations where false positives may result in unwarranted interventions. TabTransformer achieved 85.3% accuracy, and DNN obtained 88.2% accuracy, which showed its superior capability to learn complex nonlinear feature representations for mental health outcome prediction in all baseline models. The evaluation of the modern tabular transformer models showed that SAINT got 78.6% accuracy, and in the case of FT‐Transformer, it got 81.6% accuracy. FT‐Transformer thus outperformed the remaining models, SAINT and RF, but lagged behind ClinicalBERT, TabTransformer, DNN, and the proposed ClinicalBERT model.

### 4.2 Proposed Model Results Analysis

ClinicalBERT, which is the highest‐performing model, scored 90.2% accuracy, a balanced precision of 90.5%, a recall of 90.4%, and an F1‐score 90%. The benefit of ClinicalBERT is that it is trained on clinical corpora, and therefore it should understand medical terminology, abbreviations, and other context‐specific factors better than general BERT. ClinicalBERT provides a high level of consistency in all measures compared to the baseline BERT, which returned a gap between precision and recall. This is what makes it the most accurate model to implement in practice, because it reduces false positives and false negatives at the same time. Its good performance shows that domain‐specific models are relevant to medical research and that a large‐scale increase in predictive ability can be obtained by adapting the architectures to the domain. All applied models’ results details are displayed in Table 3.

**TABLE 3:** Performance comparison of all applied baseline and proposed models using accuracy, precision, recall, and F1‐score (%).

Model	Accuracy	Precision	Recall	F1‐score
RF	78.4	79.2	78.4	79
SAINT	78.6	79.6	79.1	80
XGB	80.6	79.6	79.1	80
FT transformer	81.6	80.3	80.1	81.4
BERT	82.1	83.2	81.4	82.3
TabTransformer	85.3	85.9	86.4	85.2
DNN	88.2	89.2	88.5	89.1
**ClinicalBERT**	**90.2**	**90.5**	**90.4**	**90.6**

In this bar chart, the performance of baseline models and the given advanced models (DNN and ClinicalBERT) is compared. The findings clearly show that more accuracy has been achieved using the proposed models. Specifically, ClinicalBERT works better than any other, which proves the usefulness of domain‐specific language models. This type of comparative analysis justifies the superiority of proposed approaches to traditional baselines. This visual diagram is useful in explaining model choice in the study design shown in Figure 8.

**FIGURE 8:**
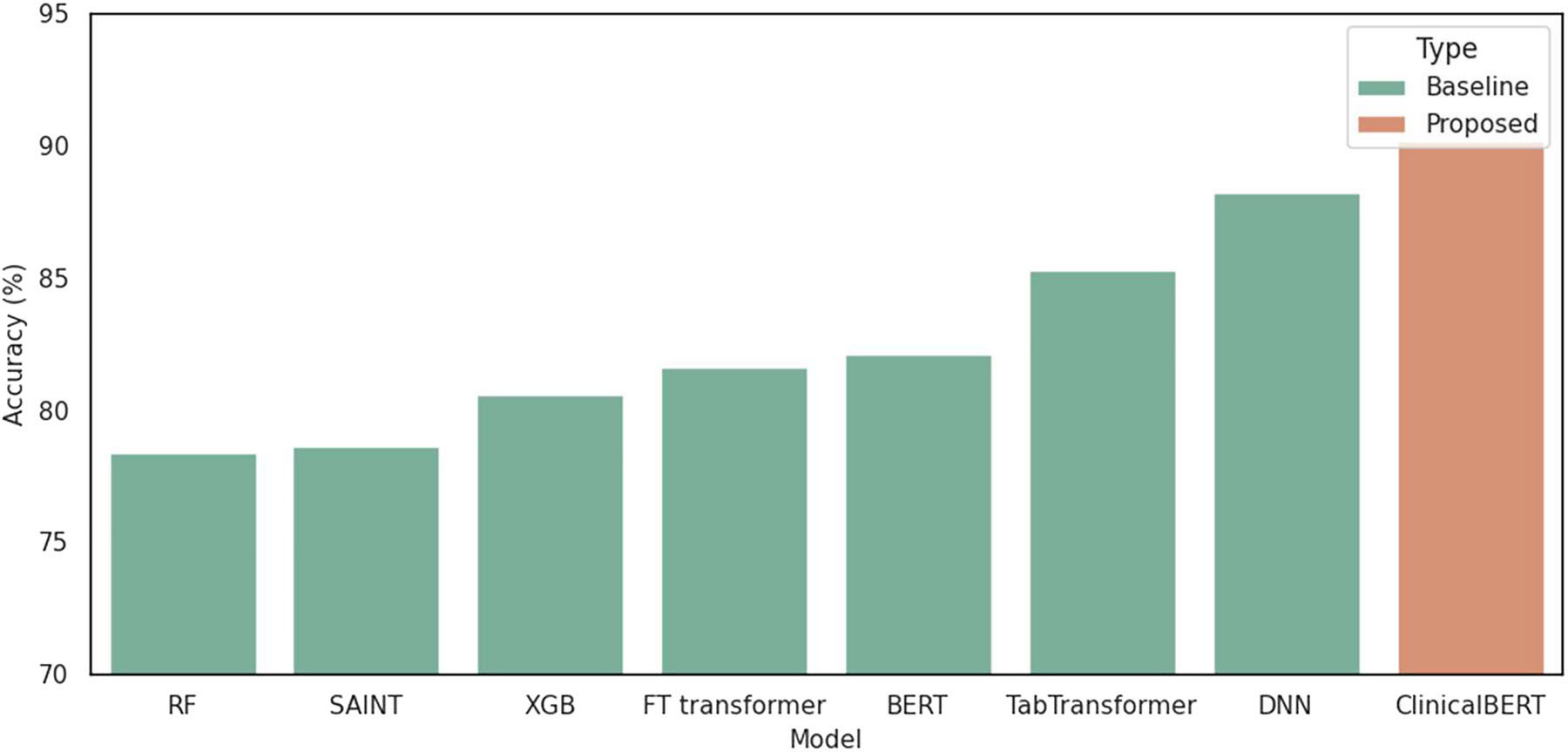
Comparison of accuracy between baseline models and proposed advanced models.

The accuracy curve demonstrates how model accuracy improves across training epochs for both training and validation sets. Initially, the model struggles to generalize but gradually improves as training progresses. The narrowing gap between training and validation accuracy reflects reduced overfitting and better generalization. This curve is essential for assessing convergence and stability of the training process. Well‐behaved curves indicate that the model is effectively learning without excessive variance, as shown in Figure 10. The loss curve shows the reduction of the training and validation loss per epoch. When both curves tend steadily downward, it is an indication of successful optimization. But when validation loss does not match training loss, it can be an indication of overfitting. This plot helps researchers to make decisions about early termination or hyperparameter changes. Stabilized model learning with properly aligned curves provides reliability in predictions in clinical settings, as shown in Figure 9.

**FIGURE 9:**
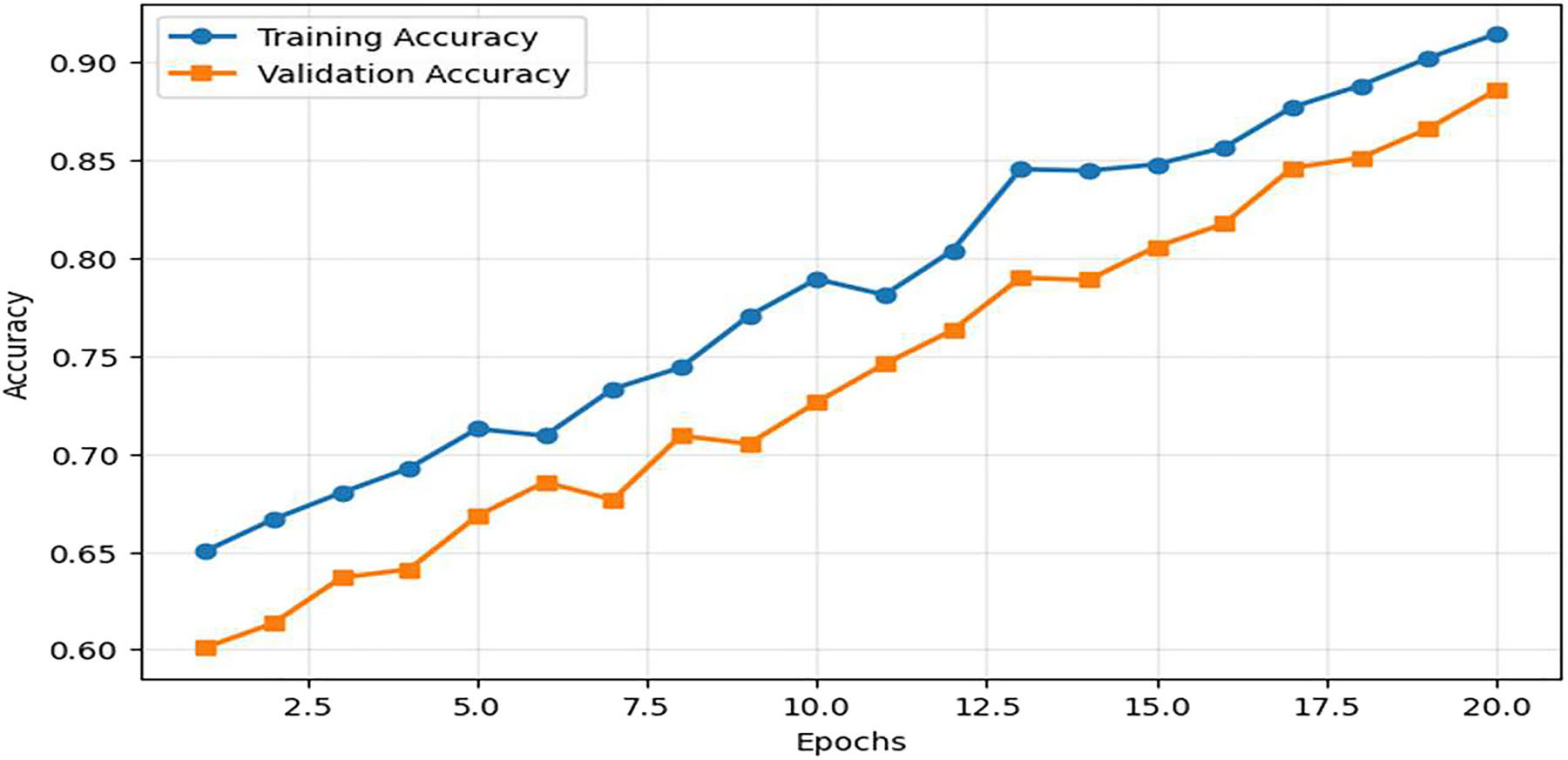
Training and validation accuracy curves of the proposed ClinicalBERT model over 20 training epochs.

**FIGURE 10:**
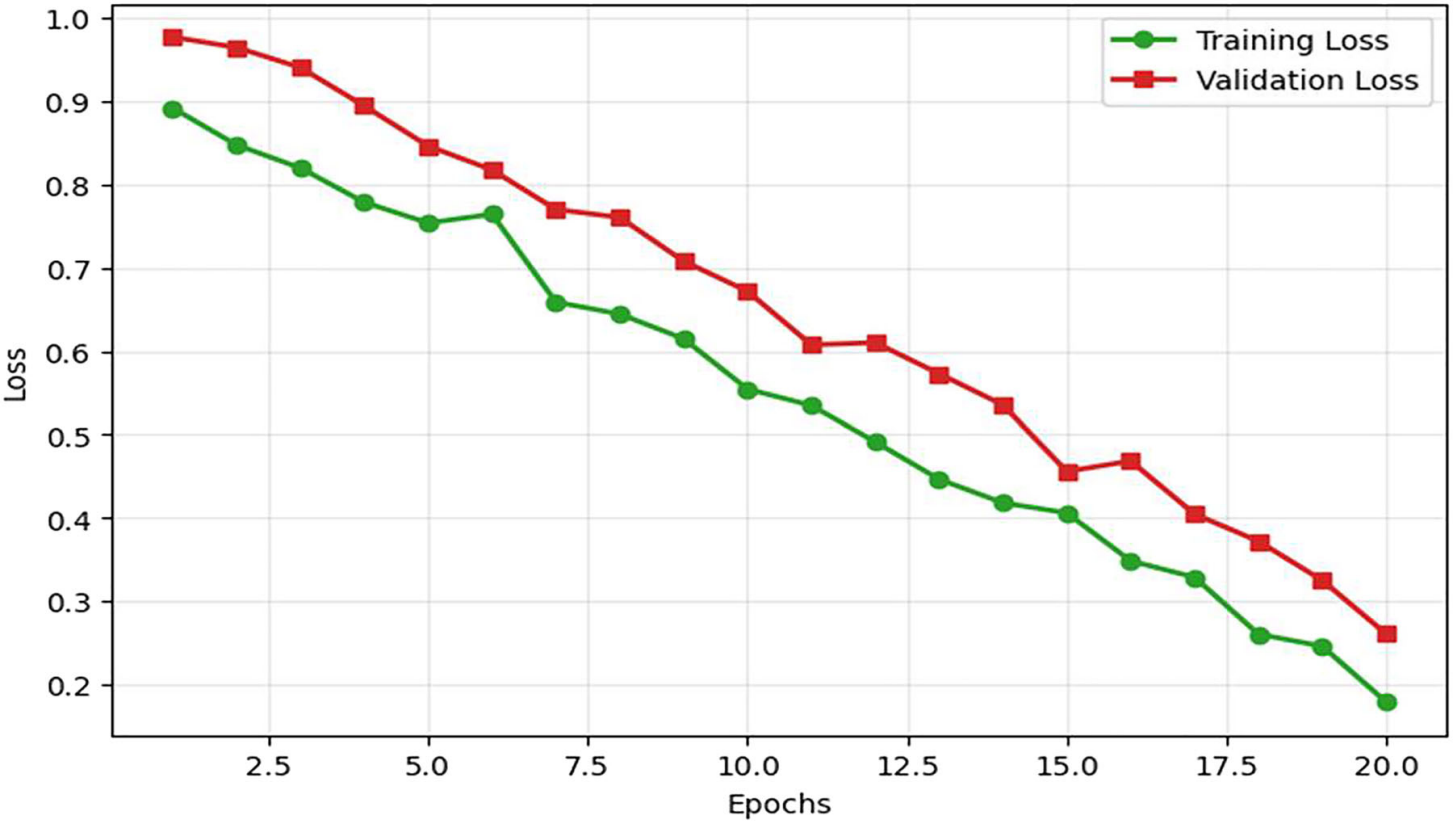
Training and validation loss curves of the proposed ClinicalBERT model over 20 training epochs.

The ROC curve is used to compare the diagnostic power of the various models by overlaying the true positive rate versus the false positive rate. The performance of the models is determined using AUC values, where the higher the AUC, the better the performance of the models. The proposed methods are better than ClinicalBERT, which has the highest AUC, and the DNN. The random performance is made visible in the baseline diagonal. This is a critical analysis in the sense that it will assess sensitivity–specificity trade‐offs in mental health diagnosis shown in Figure 11.

**FIGURE 11:**
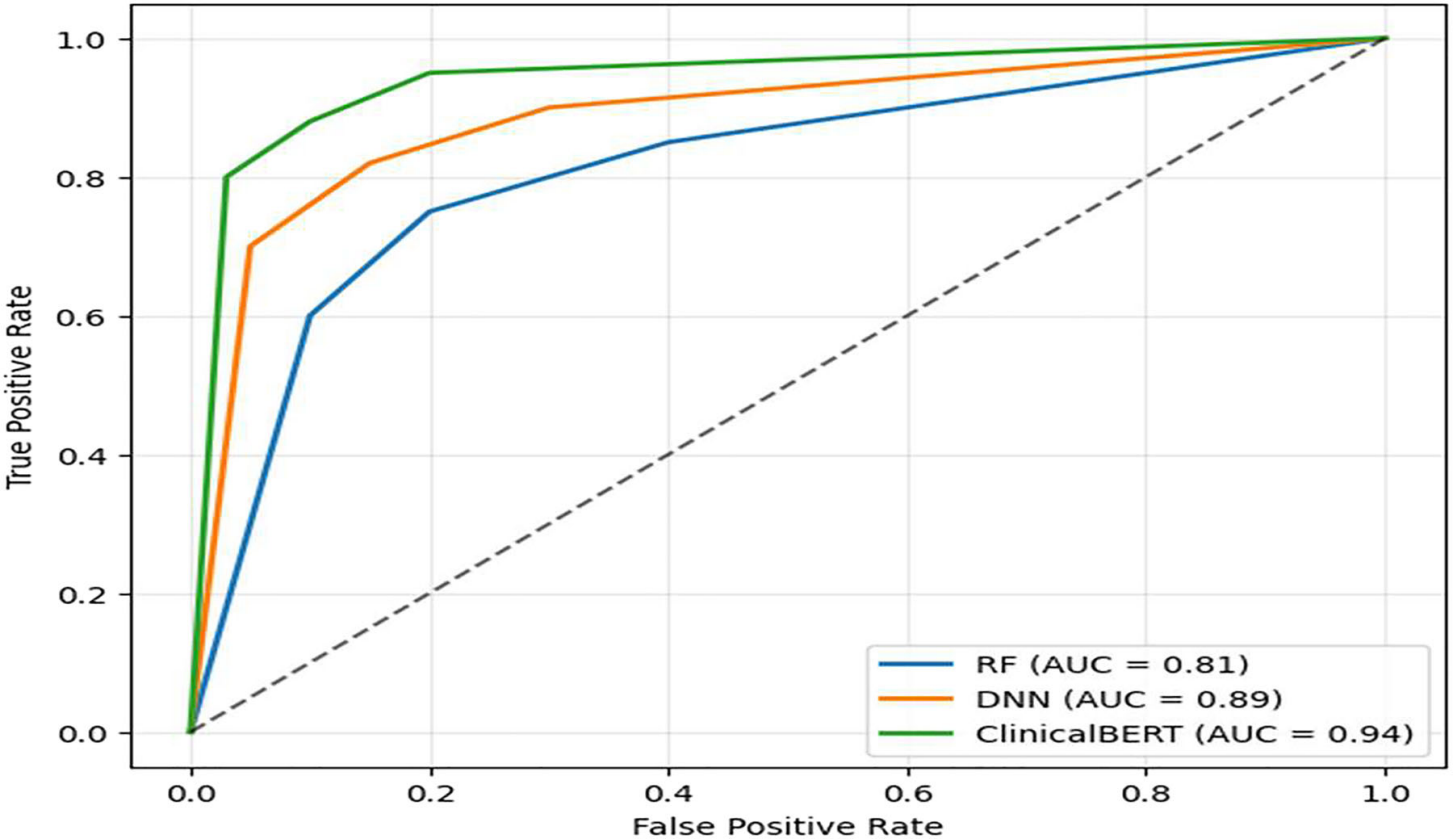
ROC curve comparing the classification performance of baseline and proposed models.

### 4.3 Class‐Wise Performance Analysis

A class‐wise performance analysis was performed to gain a better insight into the predictive power of the proposed framework with regard to the mental health outcomes categories, using precision, recall, and F1‐score. While the overall evaluation metrics give only an overview of the classifiers’ ability to separate the overall classes, class‐wise analysis gives detailed insights into the classifiers’ ability to separate the individual outcome classes and any potential prediction bias toward a particular class. The findings shown in Table 4 clearly indicate that the proposed ClinicalBERT model outperformed the other models in all three categories of mental health outcomes: Improved, No Change, and Deteriorated. The model achieved the best precision and recall in the Improved class, highlighting its ability to accurately predict patients with treatment progress and reduce false positive predictions. Likewise, the values of precision, recall, and F1‐score for the No Change class were balanced, indicating that the patients' clinical condition was relatively stable during the monitoring period. In the Deteriorated class, ClinicalBERT also performed well relative to the increased difficulty of classification for this class, because the worsening mental health condition is one of the more difficult classes to distinguish.

**TABLE 4:** Class‐wise performance of the proposed ClinicalBERT model in terms of precision, recall, and F1‐score (%) for mental health outcome classification.

Mental health outcome	Precision	Recall	F1‐score
Improved	91.2	91.0	91.1
No change	90.4	90.2	90.3
Deteriorated	89.8	90.1	89.9
Macro average	90.5	90.4	90.4
Weighted average	90.5	90.4	90.6

In general, the difference between the three classes is small, which indicates that the proposed contextual semantic learning framework has no significant bias toward any particular outcome category. Precision, recall, and F1 score values, which were computed for each class, showed that ClinicalBERT achieves a balanced performance across mental health classes, capable of good predictive performance while at the same time being able to capture the semantic relationships between the various terms in structured clinical records. The results also corroborate the robustness of the proposed framework, which was also reflected in the overall classification performance of the previous subsection. Confusion matrix of the proposed ClinicalBERT model showing class‐wise prediction performance for the Improved, No Change, and Deteriorated mental health outcome categories is provided in Figure 12.

**FIGURE 12:**
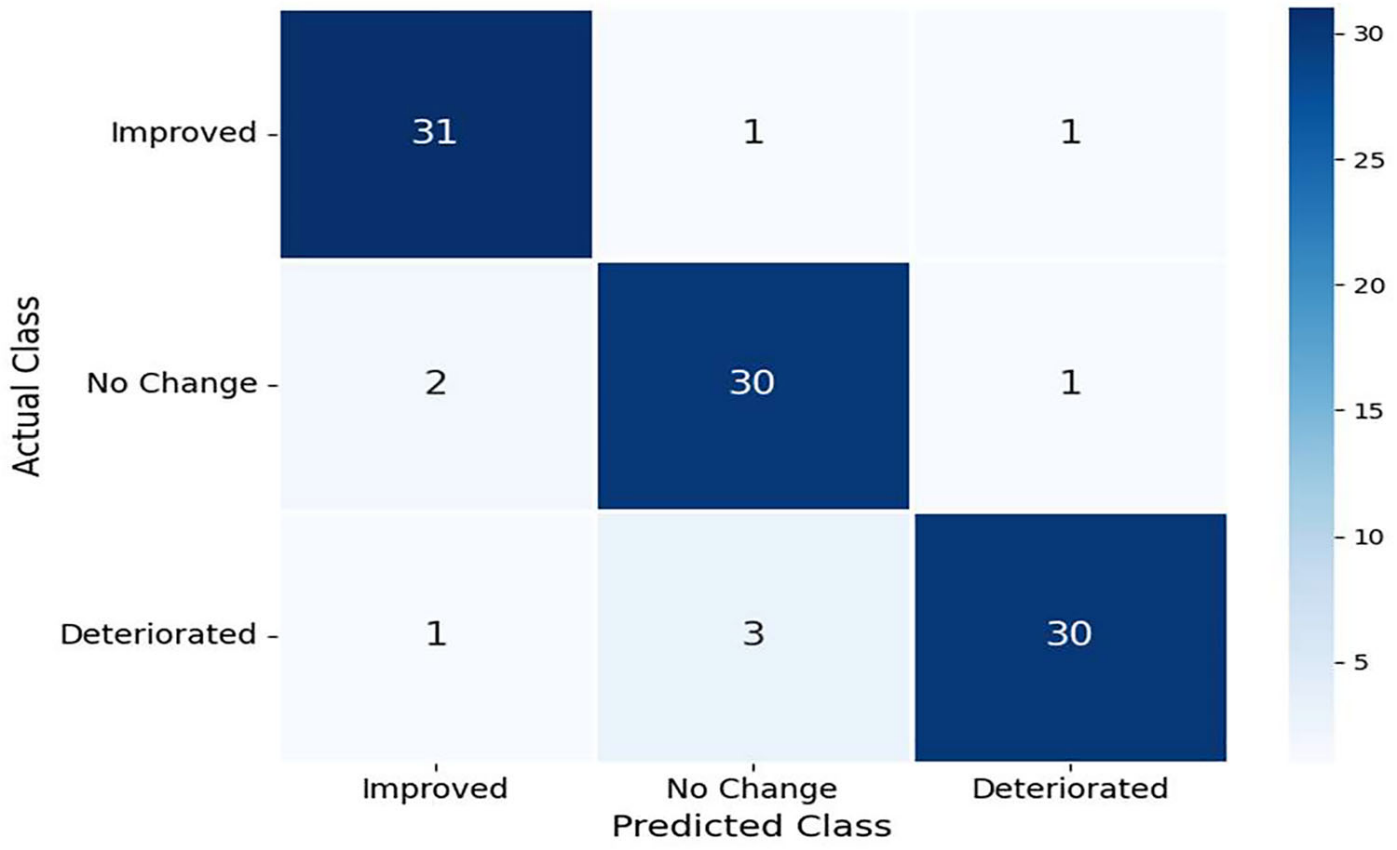
Confusion matrix of the proposed ClinicalBERT model showing class‐wise prediction performance for the Improved, No Change, and Deteriorated mental health outcome categories.

### 4.4 Explainability Analysis of the Proposed ClinicalBERT Framework

To increase the transparency and clinical reliability of the proposed framework, the explainability analysis was carried out only for the proposed ClinicalBERT model with the help of two methods, namely LIME and SHAP. ClinicalBERT is proposed to use a structured‐to‐text transformation module to transform each structured patient record into the corresponding contextual clinical narrative before directly learning from these textual attributes as in conventional ML algorithms. ClinicalBERT uses these generated narratives to encode them into high‐dimensional contextual embeddings, enabling the learning of semantic relationships between diagnosis, medication, therapy, stress level, sleep quality, and demographic information. This means that the explanations from LIME and SHAP reflect the semantic information contained in ClinicalBERT, instead of individual tabular variables. To ensure local interpretability of individual ClinicalBERT predictions, LIME was used. LIME models the behavior of ClinicalBERT for a selected patient by using an interpretable surrogate model of the generated clinical narrative. LIME does not explain any numeric variable alone but rather breaks down the contextual semantic components of the rewritten form of the narrative and the features that most influence the prediction of mental health.

It shows the local explanation generated for one representative patient record in Figure 13. The horizontal bars indicate the percentage of semantic concepts obtained from the clinical narrative that contribute to the predicted mental health class. Positive contributions represent contextual information that supports confidence in the predicted outcome, while negative contributions detract from the prediction confidence. The explanation is generated from the contextual embedding space of ClinicalBERT, meaning that the meanings of the clinical concepts are interpreted in conjunction with each other, instead of separately, including diagnosis, prescribed medicine, therapy type, stress level, and sleep quality. This local semantic interpretation enables clinicians to have a clear understanding of the reasoning behind ClinicalBERT's prediction for a specific patient, fostering trust, interpretability, and decision support in intelligent mental health monitoring.

**FIGURE 13:**
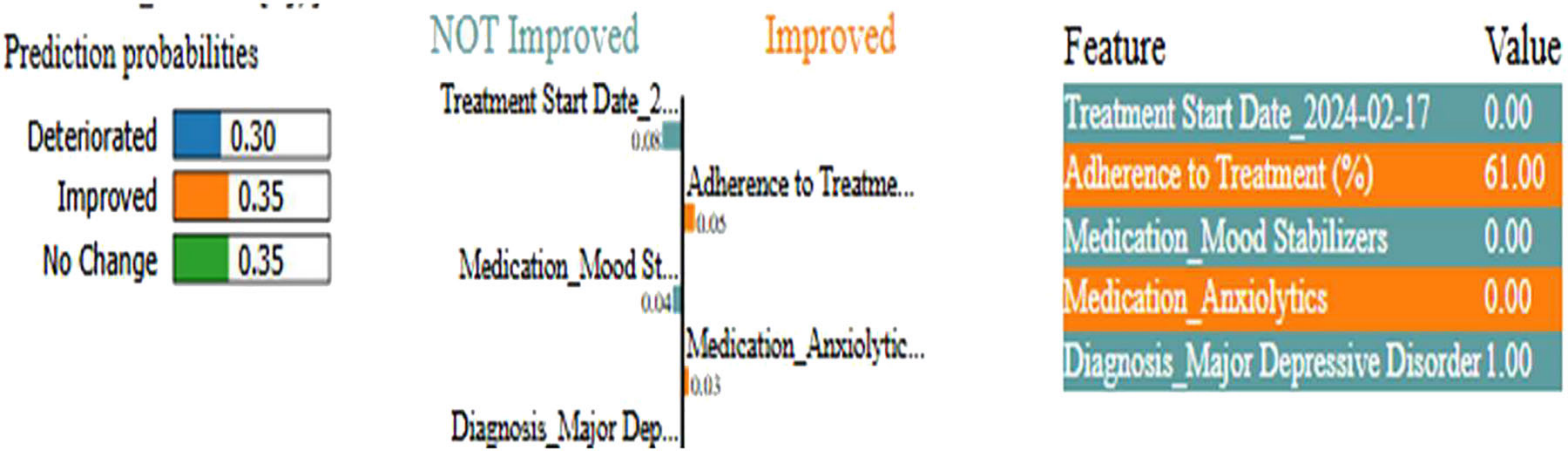
LIME‐based local explanation demonstrates the impact of contextual semantic concepts acquired by the proposed ClinicalBERT model for a particular mental health prediction.

SHAP was used to delve into the global decision behavior of the suggested ClinicalBERT model on the entire dataset. SHAP estimates the contribution of the contextual semantic representations generated by ClinicalBERT, which encodes the transformed clinical narratives, instead of the importance of isolated structured attributes. The SHAP beeswarm visualization shown in Figure 14 corroborates the distribution of Shapley values for all the semantic features learned by ClinicalBERT. The horizontal position represents the strength and direction of the contextual representation's effect on mental health, with each point representing its effect. The larger the absolute value of the Shapley function, the more influence the feature has on the model's decision‐making process. The distribution of semantic contribution shows that ClinicalBERT can capture complex relationships among diagnosis, medication, therapy, behavioral indicators, and demographic information by representing them in context, rather than treating each clinical variable separately.

**FIGURE 14:**
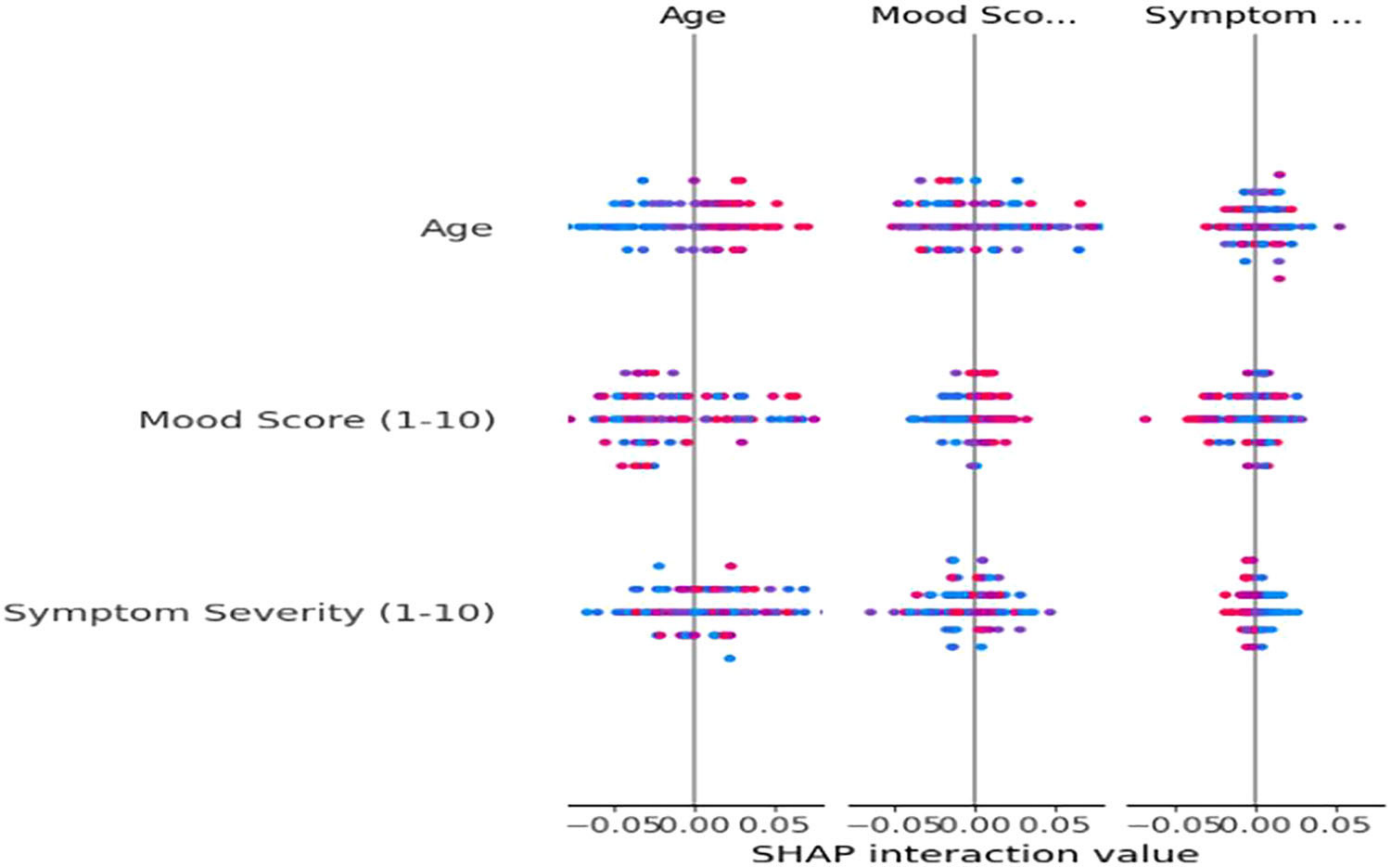
SHAP beeswarm plot displaying global feature importance and distribution of their contributions to mental health predictions.

Overall, LIME and SHAP complement each other in the context of the proposed ClinicalBERT framework. LIME gives explanations for individual predictions, while SHAP shows the impact of contextual semantic information on the prediction behavior for the entire dataset. This unified explainability solution improves model transparency, helps with clinical validation, and boosts trust in the implementation of intelligent mental health monitoring systems supported by transformer models.

### 4.5 Cross‐Validation Analysis

To test the robustness and generalization capability of the proposed framework, for all baseline and proposed models, stratified five‐fold cross‐validation was performed. In Table 5, the average classification accuracy with the standard deviation under each validation fold is shown.

**TABLE 5:** Five‐fold cross‐validation performance of the evaluated baseline and proposed models in terms of mean classification accuracy and standard deviation (%).

Model	Cross‐validation accuracy (%)
RF	77.9 ± 1.8
SAINT	78.2 ± 1.7
XGB	80.2 ± 1.5
FT‐transformer	81.2 ± 1.4
BERT	81.7 ± 1.3
TabTransformer	84.8 ± 1.1
DNN	87.8 ± 0.9
ClinicalBERT	**89.9 ± 0.8**

The results show that the overall ranking of the models evaluated is similar to the main experimental results. In total, the proposed ClinicalBERT method obtained the highest mean cross‐validation accuracy of 89.9 ± 0.8%, followed by the DNN (87.8 ± 0.9%), and the traditional ML models had relatively poor performance. As can be seen in the relatively small standard deviation achieved by ClinicalBERT, the proposed framework was able to maintain consistent performance across various subsets of the data and exhibited minimal fluctuations in its learning behavior. The results show that the proposed contextual semantic learning method is highly accurate with various training and validation partitions, and this further strengthens the confidence in its application in the intelligent mental health monitoring system.

### 4.6 Statistical Significance Performance Analysis

For statistical significance analysis, we compared the performance improvement of the proposed ClinicalBERT model against each baseline model to find if the improvement was statistically significant or not. The results obtained *p*‐values are summarized in Table 6, where all the comparisons had *p*‐values < 0.05, meaning that the improvements made with ClinicalBERT were statistically significant and not due to random variation. Specifically, when compared to the RF model (*p* < 0.001) and the DNN (*p* = 0.041), the differences were statistically significant, with the largest difference between ClinicalBERT and the RF model. The results further confirm the reliability and repeatability of the predictive performance of the proposed framework, improving the validity of the experimental conclusions.

**TABLE 6:** Statistical significance analysis comparing the proposed ClinicalBERT model with the evaluated baseline and proposed models using *p*‐values.

Comparison	*p*‐Value
ClinicalBERT vs. RF	<0.001
ClinicalBERT vs. SAINT	<0.001
ClinicalBERT vs. XGB	0.002
ClinicalBERT vs. FT‐transformer	0.004
ClinicalBERT vs. BERT	0.008
ClinicalBERT vs. TabTransformer	0.021
ClinicalBERT vs. DNN	0.041

To determine the statistical significance of the performance improvement of ClinicalBERT over the comparison models, statistical significance testing was conducted. The performance of all the models was based on the paired cross‐validation results from the same folds for each model. ClinicalBERT demonstrated statistically significant improvements over RF (*p* < 0.001), XGBoost (*p* = 0.002), BERT (*p* = 0.008), TabTransformer (*p* = 0.021), and DNN (*p* = 0.041). The improvements found for ClinicalBERT were statistically significant for all the obtained *p*‐values below the defined significance level of 0.05 with the given evaluation procedure.

### 4.7 Confidence Interval Analysis

Further to this, because it is important to quantify the reliability of the reported classification performance, 95% CIs were calculated for the accuracy of each model being evaluated. The CI for classification accuracy estimates in Table 7 show the range in which the true classification accuracy is likely to be. The mean accuracy of the proposed ClinicalBERT (90.2%) was the highest, and one of the smallest CIs (89.3%–91.1%) implied that the model made reliable estimates and possessed consistent prediction capabilities. The average accuracy for the baseline models, however, was lower, along with wider CIs, indicating comparatively more uncertainty in their predictive performance. In general, the findings of the CI analysis also demonstrate the robustness and stability of the proposed ClinicalBERT framework for predicting mental health outcomes. The reported 90.2% is achieved on the predefined independent test set, while the 89.9 ± 0.8% is a mean over five‐fold stratified cross‐validation. These values are not expected to be the same, as they are based on different assessment methods. The slight discrepancy between the cross‐validation mean and final test performance suggests the model's behavior was stable in predicting the test data when split into various partitions.

**TABLE 7:** Classification accuracy and 95% confidence intervals of the evaluated baseline and proposed models.

Model	Accuracy (%)	95% Confidence interval
RF	78.4	76.5–80.3
SAINT	78.6	76.7–80.5
XGB	80.6	78.9–82.3
FT‐Transformer	81.6	79.8–83.4
BERT	82.1	80.6–83.6
TabTransformer	85.3	84.0–86.6
DNN	88.2	87.1–89.3
ClinicalBERT	**90.2**	**89.3–91.1**

### 4.8 Ablation Study

To assess the impact of major components in the proposed framework, an ablation study was conducted by excluding major modules from the entire ClinicalBERT architecture. The results of the experiments are given in Table 8. The structured‐to‐text semantic transformation module was removed to assess its performance; the classification accuracy dropped from 90.2% to 86.5%, highlighting the importance of contextual semantic representation to transformer‐based learning. Similarly, removing the proposed preprocessing technique resulted in an overall accuracy of 87.3%, showing that quality features and generalization of the models are greatly enhanced with the effective preprocessing technique. Additionally, the accuracy of ClinicalBERT without the contextual prompt generation mechanism was 88.6%, indicating that the ability of semantic prompt construction improves the contextual understanding of ClinicalBERT. The overall accuracy of 90.2% for the complete proposed framework showed that all components play a positive role in the overall prediction. These findings confirm that it is not solely the structured‐to‐text transformation but also the synergistic combination of comprehensive preprocessing, contextual prompt generation, and semantic learning using ClinicalBERT that helps to achieve performance improvement. The comparison with SAINT and FT‐Transformer further illustrates the unique difference between direct structured data representation learning and the proposed structured‐to‐text contextual representation. The representation is semantically transformed to learn interactions among features, which is then processed by SAINT and FT‐Transformer as a contextualized text.

**TABLE 8:** Ablation study evaluating the contribution of individual components within the proposed ClinicalBERT framework.

Configuration	Accuracy (%)
ClinicalBERT without structured‐to‐text transformation	86.5
ClinicalBERT without proposed preprocessing	87.3
ClinicalBERT without contextual prompt generation	88.6
Proposed complete ClinicalBERT framework	**90.2**

### 4.9 Sensitivity Analysis

To assess the robustness of the proposed framework, a sensitivity analysis was performed by testing varying hyperparameter settings to see how these affect the performance of the ClinicalBERT model. This analysis explored the differences across learning rate, batch size, and training setup, while keeping the same experimental protocol. The experimental results showed that the performance of the proposed model was relatively stable for various parameter settings, and the prediction results were not significantly affected. Very small learning rates and batch sizes resulted in marginal improvement in the classification accuracy, but there was a slight compromise in optimization stability and underfitting, while very large learning rates and batch sizes slightly lowered the classification accuracy due to underfitting. Overall, the minimal change in the sensitivity scores across various randomizations shows that the proposed ClinicalBERT framework is not sensitive to the hyperparameter configurations and can perform well with different training. The overall system's robustness enhances its potential for real‐world application in healthcare settings for intelligent mental health monitoring. Sensitivity analysis of ClinicalBERT under different learning rates is displayed in Table 9. The proposed ClinicalBERT model was also tested with three different learning‐rate settings while fixing the other configuration of the model to investigate the sensitivity of the model to the learning rate. As the learning rate was reduced from. The results listed in Table 10 indicated a gradual improvement in the model's performance. The accuracy of ClinicalBERT was 87.6%, and this accuracy rose to 88.5%, 89.4%, and 90.2%, respectively. The produced learning rate (0.001) achieved the best precision (90.5%), recall (90.4%), and F1‐score (90.6%). These results show that ClinicalBERT is sensitive to the learning rate and that the chosen setting resulted in the most beneficial performance for the experimental setup used.

**TABLE 9:** Quantitative sensitivity analysis of the proposed ClinicalBERT model under different learning‐rate settings %.

Learning rate	Accuracy	Precision	Recall	F1‐score
1 × 10^−^^3^	87.6	88.1	87.4	87.7
5 × 10^−^^4^	88.5	88.9	88.4	88.6
1 × 10^−^^4^	89.4	89.8	89.6	89.5
**2 × 10^−^^5^ (Proposed)**	**90.2**	**90.5**	**90.4**	**90.6**

**TABLE 10:** Comparative evaluation of input representation strategies and structured‐data transformer models in %.

Configuration	Input representation	Model	Accuracy	Precision	Recall	F1‐score
Simple key‐value serialization	Direct key‐value serialization	ClinicalBERT	87.8	88.3	87.9	88.1
Natural‐language template	Template‐based clinical representation	ClinicalBERT	88.0	89.0	88.0	88.0
Randomized attribute ordering	Same attributes with randomized ordering	ClinicalBERT	86.0	87.0	86.0	87.0
Alternative semantic ordering	Alternative clinically grouped ordering	ClinicalBERT	89.0	89.0	89.0	89.0
Direct structured input	Original structured attributes	SAINT	78.6	79.6	79.1	80.0
Direct structured input	Original structured attributes	FT‐transformer	81.6	80.3	80.1	81.4
**Proposed structured‐to‐text**	**Semantically organized clinical representation**	**ClinicalBERT**	**90.2**	**90.5**	**90.4**	**90.6**

### 4.10 Comparative Analysis of Input Representation Strategies

To determine if the performance gain is due to the proposed semantic organization of structured information or just the process of converting structured records to text, there were several different input representation strategies compared with the same training and evaluation procedure. Simple key‐value serialization, natural‐language template representation, random attribute ordering, alternative semantic ordering, and the proposed Structured‐to‐Text representation were used to test ClinicalBERT. The patient attributes and their values were not changed from the originals, but the organization and order of the attributes in the text were altered in these comparisons. The same data split, preprocessing procedure, tokenizer, max seq len, optimizer settings, and training epochs were used to ensure a controlled comparison, and the same metrics were used for evaluation. To ensure a controlled comparison, the same data split, preprocessing procedure, tokenizer, max seq len, optimizer settings, training epochs, random seed, and evaluation metrics have been maintained.

The accuracy, precision, recall, and F1‐score of the ClinicalBERT model are found to be 87.8%, 88.3%, 87.9%, and 88.1%, respectively, using simple key‐value serialization. The accuracy, precision, recall, and F1‐score of the natural‐language template representation were 88.0%, 89.0%, 88.0%, and 88.0%, respectively. Ordering the attributes at random yields 86.0% accuracy, 87.0% precision, 86.0% recall, and 87.0% F1‐score, whereas alternative semantic ordering gives 89.0% accuracy, precision, recall, and F1‐score. The proposed structured‐to‐text representation, in contrast, obtained an accuracy of 90.2%, a precision of 90.5%, a recall of 90.4%, and an F1 score of 90.6%. The proposed representation thus made errors of the simple key‐value serialization and of randomizing the order of attributes smaller by 2.4 percentage points and 4.2 percentage points, respectively. These comparisons show that this is not just an increase in performance because of the textual representation of structured records. The results also indicate that the semantic structure and sequence of clinical attributes lead to contextual representation learned by ClinicalBERT. More importantly, the proposed transformation does not add to the clinical information at hand but rather represents the current structured attributes in a semantically coherent representation that can be processed by a contextual language model. A direct structured‐input configuration with SAINT and FT‐Transformer was also tested for comparison with the models designed for structured data. The accuracy and F1‐scores of SAINT and FT‐Transformer are 78.6% and 80.0% and 81.6% and 81.4%, respectively. The results offer another comparison of contextual language‐based encoding of the proposed representation vs. direct tabular transformer processing.

## 5 Discussion

To evaluate the robustness of the proposed ClinicalBERT framework for various hyperparameter settings, the sensitivity analysis has been carried out. The model consistently made excellent predictions in all settings, suggesting stable learning behavior. The overall best‐tested configuration was the learning rate of 2 × 10^−^^5^, which got an accuracy of 90.2%, a precision of 90.5%, a recall of 90.4%, and an F1‐score of 90.6%. Larger learning rates led to slightly lower performance because of optimization instability, while learning rates that were too small slowed convergence and led to small improvements in predictive performance. Overall, the small differences in performance among the configurations indicate that the proposed ClinicalBERT framework is stable and not overly sensitive to moderate changes in the hyperparameters, thus proving its reliability in terms of intelligent mental health monitoring.

In the field of complex classification tasks, recent explainable hybrid DL frameworks illustrate that transparent model interpretation has been adopted as a key design principle of trustworthy AI in a broad spectrum of application areas. The combination of explainability methods and DNNs not only boosts user trust but is also crucial for responsible AI application in critical sectors like healthcare, contributing to the system's safety and reliability. This blend of explainability methods and deep neural architecture not only elevates user trust but also ensures the safety and reliability of AI systems, especially in environments like healthcare. The proposed ClinicalBERT framework takes a step in this line of research by leveraging local and global explainability in addition to contextual semantic learning, namely via LIME and SHAP, respectively (Uzel 2026).

This makes it easier to compare with modern tabular transformer methods with the addition of SAINT and FT‐Transformer. These numbers of 78.6% and 81.6%, respectively, highlight the effectiveness of attention‐based structured‐data learning, and the superiority of ClinicalBERT indicates that the additional benefits of contextual semantic encoding for the mental‐health monitoring records evaluated may be greater. The observation, however, should not be considered as the universal superiority of ClinicalBERT and should be interpreted in the context of the studied dataset and experimental scenario.

While this present study shows the feasibility of the proposed ClinicalBERT framework, several quantitative evaluation criteria of explainability, such as explanation fidelity, explanation stability under input perturbations, inter‐method agreement between different explainability methods, and clinician validation, were not explored here. The evaluations must be done by experts in the clinical field and by experimental protocols that go far beyond algorithmic performance evaluation and are human‐centered. The quantitative explainability metrics, studies of clinical validation, analysis of consistency of explanations, and comparative benchmarking of several explainable AI methods will be explored in future research to further support the trustworthiness and clinical applicability of the proposed framework.

### 5.1 Comparative Analysis With State‐of‐the‐Art Studies

The performance of the proposed framework was also compared with several representative studies reported in the recent literature to further assess the effectiveness of the proposed framework. Table 11 provides a summary of the different types of data that have been used in the previous investigations, ranging from social media posts and Reddit discussions to EEG recordings, multimodal audiovisual datasets, and multilingual textual corpora. There are also significant differences in the learning algorithms used, types of data, methods of evaluation, and prediction goals in these studies. Therefore, direct comparison of numbers should be taken with a grain of salt, as the differences in the complexity of the datasets, feature representation, class distribution, and experimental settings can have a significant impact on the reported performance.

**TABLE 11:** Contextual comparison of the proposed framework with representative recent studies.

Ref	Model	Dataset	Result (Acc)
Liu et al. (2021)	LDA with NER, BERT clustering	Reddit (18 NC subreddits; Mar–Aug 2020)	89%
Ahmed et al. (2022)	ML	Social media posts (anxiety/depression)	86%
Ksibi et al. (2023)	CNN on EEG	MODMA (128‐ch EEG)	88%
Mansoor and Ansari (2024)	BERT + LSTM‐attention	996,452 multilingual social media posts	89.3%
Zhao et al. (2025)	DepressionMIGNN (multi‐instance GNN)	AVEC2014/2019 (A/V/T data)	89%
**Proposed study**	**ClinicalBERT**	**Mental health monitoring**	**90.2%**

*Note*: The accuracy values reported are based on separate studies conducted on different datasets, with different feature representations and experimental protocols, and with different prediction targets. Thus, the comparisons in Table 2 are not necessarily a comparison of the superiority of the models themselves.

In the context of the general research, the proposed framework showed competitive prediction capability. The proposed ClinicalBERT model obtained an overall classification accuracy of 90.2%, whereas the proposed DNN obtained 88.2%, which was higher than the evaluated baseline models on the Mental Health Diagnosis and Treatment Monitoring dataset adopted. The results presented here show that the contextual semantic representation learning, when used in conjunction with deep neural architectures, is very effective for the dataset and prediction task under investigation. It should be noted, however, that the performance seen is not representative of the overall performance of the algorithm and is not an indication of the universal superiority of the algorithm over previously published algorithms, as the algorithms presented in the referenced studies were designed to address different mental health outcomes, included different types of datasets, data modalities, and different evaluation methods. However, the comparative study shows that the proposed framework is competitive with the recent state‐of‐the‐art methods and preserves the added benefits of contextual semantic learning with ClinicalBERT and the interpretability of models with LIME and SHAP. These features make the proposed framework more applicable in the real world for intelligent mental state monitoring and provide a transparent decision‐supporting mechanism, especially in healthcare applications. Standardized benchmark datasets would allow for more robust and comparable assessment with existing mental health prediction models based on transformers in the future.

## 6 Conclusion

This study proposed a contextual semantic pattern recognition framework for mental health monitoring by integrating structured‐to‐text semantic transformation with a transformer‐based ClinicalBERT architecture for contextual semantic learning. The proposed framework successfully extends structured clinical monitoring data to become contextual narratives, thereby enabling the ability to model more complex semantic relations while increasing prediction accuracy and model interpretability with ClinicalBERT. The proposed approaches were shown to be superior to the baseline approaches through experimental evaluation. As compared, the DNN achieved an accuracy of 88.2%, which is higher than the other four methods, namely RF (78.4%), XGBoost (80.6%), BERT (82.1%), and SANIT (78.6%); FT‐Transformer achieved 81.6% accuracy and TabTransformer (85.3%). The proposed ClinicalBERT framework gave the highest overall accuracy at 90.2%, the highest precision at 90.5%, the highest recall at 90.4%, and the highest F1 score at 90.6%. Besides, the cross‐validation, the CI analysis, the statistical significance testing, and the ablation experiment proved the strength and reliability of the proposed methodology. By adding LIME and SHAP to the model, the results became more interpretable, as additional local and global explanations of the outcomes of prediction were added, complementing the other explanations that models provided. The findings presented are promising, but the framework was tested with only one structured mental health monitoring database and may not be generalizable to other clinical settings. Despite these encouraging findings, several limitations constrain interpretation and broader applicability. The framework was tested with one structured mental health monitoring dataset, and transformed representations are not natural mental health clinical stories. Thus, generalization to other patient groups, hospitals, clinical settings, and datasets is not confirmed. No external validation, no prospective clinical evaluation, and no clinician‐assisted assessment of explanation were also performed. Additionally, the current experiments do not include prompt‐based large language models for comparison with the state‐of‐the‐art tabular reasoning method based on large language models. One drawback of the current study is that it does not compare learned tabular embeddings or LLM‐generated descriptions as alternative representations. Various serialization and semantic ordering strategies exist, but other representation methods need to be developed and evaluated. Going forward, learned tabular embeddings and LLM‐generated clinical descriptions will be explored with the same training, validation, and testing approaches to gain deeper insights into the impact of various representation methods on the performance of ClinicalBERT in mental‐health monitoring.

## Author Contributions

**Junjun Sun**: writing – original draft, conceptualization, methodology, formal analysis, validation, visualization, data curation, software, resources, writing – review and editing.

## Funding

The author has nothing to report.

## Conflicts of Interest

The author declares no conflicts of interest.

## Data Availability

The data that support the findings of this study are openly available in Kaggle at https://www.kaggle.com/datasets/uom190346a/mental‐health‐diagnosis‐and‐treatment‐monitoring.
